# Multiplex Eukaryotic Transcription (In)activation: Timing, Bursting and Cycling of a Ratchet Clock Mechanism

**DOI:** 10.1371/journal.pcbi.1004236

**Published:** 2015-04-24

**Authors:** Katja N. Rybakova, Frank J. Bruggeman, Aleksandra Tomaszewska, Martijn J. Moné, Carsten Carlberg, Hans V. Westerhoff

**Affiliations:** 1 Molecular Cell Physiology, VU University Amsterdam, Amsterdam, The Netherlands; 2 Systems Bioinformatics, VU University Amsterdam, Amsterdam, The Netherlands; 3 School of Medicine, Institute of Biomedicine, University of Eastern Finland, Kuopio, Finland; 4 Manchester Centre for Integrative Systems Biology, University of Manchester, Manchester, United Kingdom; 5 Synthetic Systems Biology, Netherlands Institute for Systems Biology, University of Amsterdam, Amsterdam, The Netherlands; Ottawa University, CANADA

## Abstract

Activation of eukaryotic transcription is an intricate process that relies on a multitude of regulatory proteins forming complexes on chromatin. Chromatin modifications appear to play a guiding role in protein-complex assembly on chromatin. Together, these processes give rise to stochastic, often bursting, transcriptional activity. Here we present a model of eukaryotic transcription that aims to integrate those mechanisms. We use stochastic and ordinary-differential-equation modeling frameworks to examine various possible mechanisms of gene regulation by multiple transcription factors. We find that the assembly of large transcription factor complexes on chromatin via equilibrium-binding mechanisms is highly inefficient and insensitive to concentration changes of single regulatory proteins. An alternative model that lacks these limitations is a cyclic ratchet mechanism. In this mechanism, small protein complexes assemble sequentially on the promoter. Chromatin modifications mark the completion of a protein complex assembly, and sensitize the local chromatin for the assembly of the next protein complex. In this manner, a strict order of protein complex assemblies is attained. Even though the individual assembly steps are highly stochastic in duration, a sequence of them gives rise to a remarkable precision of the transcription cycle duration. This mechanism explains how transcription activation cycles, lasting for tens of minutes, derive from regulatory proteins residing on chromatin for only tens of seconds. Transcriptional bursts are an inherent feature of such transcription activation cycles. Bursting transcription can cause individual cells to remain in synchrony transiently, offering an explanation of transcriptional cycling as observed in cell populations, both on promoter chromatin status and mRNA levels.

## Author Summary

Transcription initiation is an important process that contributes to determining mRNA and eventually protein levels. In multicellular organisms transcription activity of a single gene is regulated by many different signals. This leads to multiple transcription factors binding to the same promoter. Here we study fundamental aspects of this regulation. We show that the formation of a single regulating protein complex, consisting of tens of proteins is a very inefficient mechanism: it is slow and hard to regulate. The formation of small complexes, each leaving a histone modification on the promoter solves these problems. Optimally complexes are assembled in a strict order and new histone modifications sensitize the chromatin for the formation of the next complex. This leads to a cyclic, ordered series of irreversible events that is fast and can be tightly regulated—a cyclic ratchet mechanism; like a mechanical ‘clock’. In the ratchet model, one particular chromatin state allows for RNA polymerase rebinding, which makes bursts in mRNA production a basic feature of the ratchet mechanism. Such bursting transcriptional activity has indeed been observed for eukaryotic genes. The duration of the entire transcription cycle can easily become tens of minutes, even though single proteins reside on the chromatin for only tens of seconds. Although the formation time of protein complexes on chromatin can be highly variable, due to invariable stochasticity in protein binding and disassociation, the duration of the entire transcription cycle can be very precise. As a consequence, cells that are activated at the same moment in time display synchronous transcriptional activity for several transcription cycles. This provides an explanation for transient transcription cycles observed at the level of cell populations.

## Introduction

Eukaryotic transcription depends on dozens of proteins, including transcription factors (TFs), chromatin remodellers and RNA polymerase II components [1–7]. It is frequently more complex than prokaryotic transcription [8].

Even though we refer by ‘transcription regulation’ only to regulation of the transcription process itself and not to the regulation of gene expression through transcription, the complexity of eukaryotic transcription regulation is immense. It varies with gene function. ‘House-keeping’ genes maintain nucleosome-free regulatory regions permissive to transcription [9, 10]. Genes involved in developmental transitions switch between ‘OFF’ and ‘ON’ states under the control of multiple TFs [11] affected by the epigenetic state of the corresponding chromatin region [12]. Conditionally-active genes can respond to multiple TFs that activate RNA polymerase II complex assembly and modify the chromatin state of the gene’s regulatory region [13].

Regulation of gene activity involves protein complex formation on chromatin and changes in nucleosome states [14, 15]. Genome-wide analyses confirmed this for human transcription [16–18]. In the OFF state, nucleosomes often mask regulatory DNA sequences. Upon nucleosome remodelling, promoter regions become transiently accessible for TFs that, in turn, recruit RNA polymerase II to start transcription of selected genes in the context of chromatin [7]. Thus, the activity of a gene is determined by multiple stochastic events [19–21]. Protein complex assembly and DNA binding can be thermodynamically reversible, whereas other processes are Gibbs-energy-dependent, for instance requiring ATP hydrolysis, and irreversible, including covalent histone modifications, nucleosome eviction and transcription initiation. Irreversible events typically require enzymatic activity for their reversal. Irreversible events therefore lead to chromatin and nucleosome states that have a longer life-time than protein complex (dis-)assembly and protein residence times on chromatin. This may function as a molecular memory of the transcription cycle state [22].

In some cases transcription dynamics at the cell population level proceeds in an oscillatory fashion [23–28] at frequencies between 30 and 60 min [24, 27], i.e. much slower than the observed protein dwell times on chromatin of up to 1 min [29]. It is currently not understood how these fast molecular association and dissociation events cause such slow alterations in gene activity. Using a minimal model it was shown [30] that sequential assembly of protein complexes can in theory give rise to oscillatory gene activity. However, whether models with realistic kinetic parameters can also generate such slow dynamics remains unclear.

Despite many recent experimental studies, we lack a realistic, dynamic picture of transcription initiation that integrates time scales of nucleosome modifications, protein complex formation, RNA polymerase assembly and its escape from regulatory regions, mRNA elongation and mRNA-concentration dynamics. This hiatus precludes resolving a number of issues: do cyclic transcriptional mechanisms perform better than the reversible ones found in prokaryotes; and are they perhaps even essential? How well can an ordered transcription cycle be regulated by many factors in comparison to a random set of equilibrium binding events? Is irreversibility essential for transcription regulation by multiple TFs? Does such organization of transcription imply special regulatory and stochastic properties? Do the stochastic dynamics of the transcription cycle model agree with single-cell and population-level studies of transcription when realistic kinetic parameters are considered? Is the seconds-timescale of molecular events in agreement with transcriptional cycling of tens of minutes?

Using a variety of experimental data and observations, we here compare a number of transcription activation mechanisms in terms of their ability to be both fast enough and responsive to multiple factors. We show that fully reversible transcription activation mechanisms would not work for promoters regulated by many TFs, which is expected for conditionally regulated genes in mammals. Rather, a transcription activation clock model with irreversible ratchets does resolve diffusion limitations, the required multi-factorial regulation as well as the experimental observations on transcriptional cycling and bursting.

## Results

### Reversible equilibrium-binding mechanisms for gene regulation become ineffective when many regulatory factors are involved

**Fig 1:**
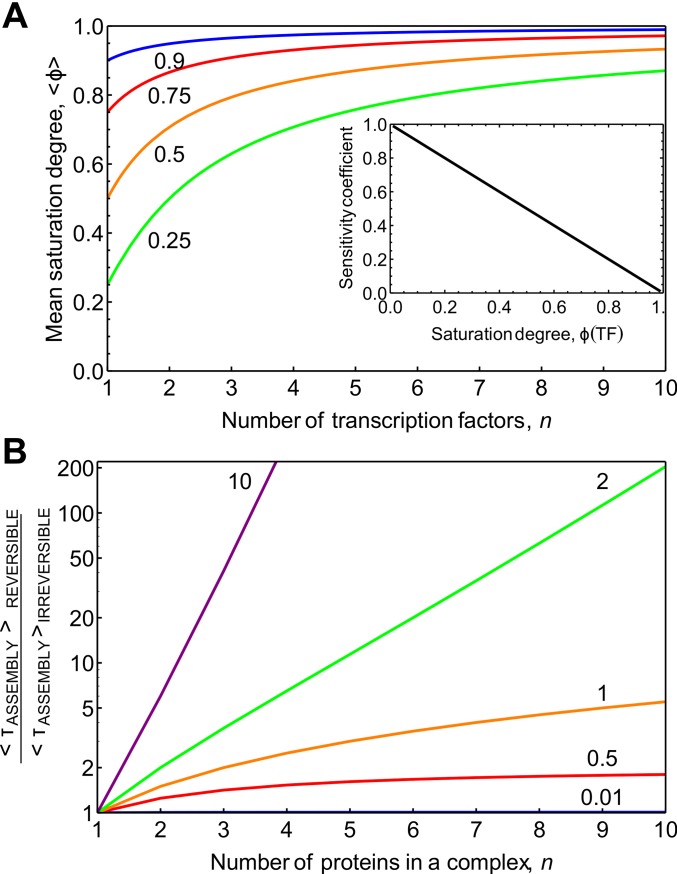
The relationship between gene saturation, sensitivity to regulation, and the dependence of protein complex assembly on reversibility. **A**. To achieve a fixed saturation degree of the entire gene (Φ(**T**, n); values considered 0.25, 0.5, 0.75, and 0.9 are indicated next to the respective lines) the required average saturation degree per TF site, ⟨*ϕ*⟩, is plotted as function of n, the number of TFs. The sensitivity of the binding sites to the TF, $R_{T_{j}}^{\Phi }$, is inversely proportional to the saturation degree as indicated in the inset. **B.** The influence of the complex size and reversibility of complex formation on the assembly time for an ordered-sequential and reversible protein complex formation mechanism. The assembly times of the reversible mechanism relative to that of the irreversible mechanism are plotted. The different lines correspond to different values for the effective dissociation constant ${\text{K}}_{\text{D}}^{\prime }$ as indicated by the numbers above the lines.

In the equilibrium-binding mechanism, TFs populate individual binding sites in a concentration-dependent manner. For convenience, we define every protein involved in transcription regulation as a TF, regardless of whether it acts merely as a scaffold, a modifier of a nucleosome or enzyme. For *n* TFs, the activity of the gene Φ(***T***), with ***T*** as the vector of TF concentrations, corresponds to the product of the saturation *ϕ*_*i*_(*T*_*i*_) of the individual, independent TF-binding sites, i.e. $\Phi \left(T\right)\equiv \frac{v}{k^{\prime }}=\prod_{i=1}^{n}\phi_{i}\left(T_{i}\right)$, with *v* as the transcription rate and *k*′ as the apparent transcription rate constant. Here *ϕ*(*T*_*i*_) denotes the saturation function of a single site, in the case of an activating TF it becomes $\frac{T_{i}}{T_{i}+K_{D,i}^{\prime }}$ and when the TF is inhibiting it equals $\frac{K_{D,i}^{\prime }}{K_{D,i}^{\prime }+T_{i}}$, with $K_{D,i}^{\prime }$ as the apparent dissociation constant of site *i* for *TF*_*i*_. Taking the product of the saturation functions of the individual, independent sites has the effect that saturating 10 activating sites by 50% leads to an activity of the gene of only 0.1% ($\Phi \left(T\right)={\left(\frac{1}{2}\right)}^{10}=\frac{1}{1024}$). In order to achieve 75% of maximal gene activity, i.e. Φ(***T***) = 0.75, each individual site would have to be virtually saturated, e.g. *ϕ*_*i*_(*T*_*i*_) = 0.98, which is unrealistic. The tendency of requiring enhanced saturation of individual binding sites is shown in Fig 1A.

Gene regulation by many regulatory factors based on equilibrium binding is also limited in terms of responsiveness. The sensitivity of the transcription rate to a specific TF depends on the extent of saturation of the gene with that TF: $\frac{\partial \;\text{ln}\;v}{\partial \;\text{ln}\;T_{i}}=1-\phi_{i}\left(T_{i}\right)=0.02$ for *ϕ*_*i*_(*T*_*i*_) = 0.98. This analysis shows that increasing the single-site saturation, which is sufficient to produce significant activation of transcription, causes the gene to loose sensitivity to regulators. Thus, in case of the equilibrium binding mechanism, genes become progressively harder to regulate when the number of regulatory factors increases. This finding is highly relevant as the number of TF-binding sites actively regulating many eukaryotic genes is readily around ten or more. A genome-wide analysis of data from ENCODE project shows in *Drosophila* that the number of functional TF-binding sites per enhancer sequence is between 2 and 15 with the average around 8 [31]. Combined evidence from several studies of individual promoter studies supports similar conclusions [32–34]. Additionally, many transcription co-factors and histone modifiers are regulated by signaling and metabolic pathways [35], increasing the number of potential regulatory inputs.

We have also considered whether cooperative binding of the TFs can overcome the deficiencies of the equilibrium-binding model (see S1 Text). We find that for a high number of TFs unrealistically high levels of cooperativity between regulatory factors are required to solve the problem of sensitivity or saturation loss (S1 Fig). We will further discuss alternative mechanisms for eukaryotic gene regulation that do not suffer from this limitation.

### Reversible assembly of large protein complexes can take tens of minutes

The mean assembly time for a protein complex of *n* factors, with identical kinetics, depends on the reversibility of complex formation ($K_{D}^{\prime }$) and the first-order association rate constant ($k_{F}^{\prime }$) as: $\tau \left(n\right)=\frac{n}{k_{F}^{\prime }}+\sum_{i=1}^{n-1}\frac{i}{k_{F}^{\prime }}{\left(K_{D}^{\prime }\right)}^{n-i}$. The irreversible assembly time equals $n/k_{F}^{\prime }$ for *n* identical factors; if factors have different association rate constants, this time is given by $\sum_{i=1}^{n}1/k_{F,i}^{\prime }$. In these equations, $k_{F}^{\prime }$ denotes the effective first order rate constant (unit: min^-1^) for the binding of a regulatory factor to a partially assembled complex. It equals the multiplication of a (diffusion-limited) second-order rate constant for binding (unit: nM^-1^ min^-1^) with the (nuclear) concentration of the regulatory factor (unit: nM).

Using these equations, one can estimate both the irreversible and the reversible assembly times of a protein complex composed out of 10 components. We take the cell nucleus volume to be 1.2 pL given that the measured cell volume is 3–4 pL [36] and that 30% of this volume is occupied by the nucleus [37]. The average copy number of sequence–specific TFs per cell, as found in whole-cell proteomic studies, is around 2500 (S2 Fig), which results in a concentration of 3.5 nM [38–41]. Similar average concentrations are found for the chromatin modifiers (S2 Fig). Protein-chromatin association rates are found to be about two orders of magnitude below the diffusion limit [42], which can be directly calculated to be around 0.01 nM^-1^s^-1^ (see S3 Table). With these numbers, $k_{F}^{\prime }$ is approximately 2 min^-1^, i.e. transcription activation could take place twice per minute if it would depend on just a single TF. For a complex of 10 TFs, assembling irreversibly in a defined sequence, assembly should then take 5 min. Ficz *et al*. [43] measured an off-rate constant (*k*_*B*_) of 2 min^-1^ for Polycomb group proteins from chromatin. This sets the dissociation equilibrium constant ($K_{D}^{\prime }$) close to 1, which is consistent with our analysis of the requirements for the promoter sensitivity to TFs. Assuming the dissociation rate constant, *k*_*B*_, to be equal to $k_{F}^{\prime }$ and substituting these numbers into the equation for *τ*(*n*), supplied above, gives for complexes of 2, 5, 7, 10, 12 and 24 TFs assembly times of 1.5, 7.5, 14, 27.5, 39 and 150 min, respectively, i.e. much longer than the times for irreversible assembly (39 versus 5 min for a complex of size 10). Notably, for the complex size of 10 the assembly time is more than twice as high as the assembly time of a complex of five proteins (7.5 min). While the exact timing of the complex assembly depends on the values of the protein copy number and the rate constants, which may vary, the overall conclusion that the reversible mechanism is considerably slower at high protein copy number is always correct for effective K_D_ around 1 or higher. These numbers indicate that protein complex formation on chromatin can become potentially rate limiting for transcription rate. The delay in reversible assembly is due to ‘hesitation’, i.e. frequent disassembly of a partially assembled complex before full assembly. Division of the reversible assembly time, *τ*(*n*), by the time for irreversible assembly of the complex, *τ*(*n*)/(*n*/*k*_*F*_), indicates the influence of reversibility and complex size on assembly time (Fig 1B): large complexes have very long assembly times and this effect becomes dramatic if the processes are more than half reversible, i.e. when the effective dissociation constant favours dissociation. For an effective dissociation equilibrium constant of 2 and an on-rate constant of 2 min^-1^ the assembly for a decameric complex is delayed by a factor of 200; i.e. the process is hesitating all the time. Then transcription activation would take 17 h rather than 5 min. This clearly points out that, if a substantial number of TFs are involved in transcription activation, the reversible mechanism may well become too slow for appropriate timing of transcription. The assembly time also depends on the precise mechanism of assembly, as discussed in S2 and S3 Texts. In particular, we find that, in general, random assembly mechanisms are faster than the sequential ones (S3 Fig) and that direct assembly on the chromatin is faster compared to the pre-assembly in the nucleoplasm (S4 Fig). In summary, our analysis indicates that for many eukaryotic genes mechanism of reversible association of all TFs becomes too slow.

### Batch assembly of partial complexes speeds up transcription initiation

Fig 1B shows that for a $K_{D}^{\prime }$ of 2, the reversible assembly of 9 TFs is 100-times slower than the irreversible assembly, whilst for a complex size of 3 it is only about 3-times slower. This suggests that a sequence of two trimeric association processes could be faster than a single 6-proteins association process. Likewise, for an effective dissociation constant of 1, the reversible continuous formation of a complex composed of 24 proteins should be 5-times slower than the corresponding assembly of 6 complexes, each of which is composed of 4 in sequence, i.e. τ(24)/6τ(4) = 5 (for *K*_*D*_ = 1 and 140 for *K*_*D*_ = 1.33). These calculations indicate that the batch-wise assembly of multi-factor complexes can reduce their otherwise long assembly times. But for this, the partial complex established in the first phase should not dissociate whilst the protein complex is being formed.

This conclusion brings us to an important finding: to be fast enough, the process of sequential formation of small protein complexes requires irreversible marking of progress (which we shall refer to as ‘ticking’), for instance, by way of covalent histone modification of nucleosomes. The transcription activation process should therefore resemble a molecular ratchet: it can ‘hesitate’ during the reversible assembly of the small protein complex but cannot return to a state where the previous set of proteins were assembling due to the histone ticking. This batch-wise mechanism has been suggested before [22] but it has never been deduced as essential for eukaryotic transcription to be fast and regulative enough (see below).

### Revertibility of transcription activation requires a cycle

One way to make the process of transcription activation revertible is to make it thermodynamically reversible. This corresponds to the equilibrium transcription activation mechanism discussed above. S5A Fig shows this mechanism for the case where 10 TFs reversibly bind to the chromatin, producing the activated transcription complex. In the S4 Text we detail why this type of mechanism and 4 others (S5 Fig), proceeding in reverse order through the transcription activation pathway, are either too slow or not revertible.

**Fig 2:**
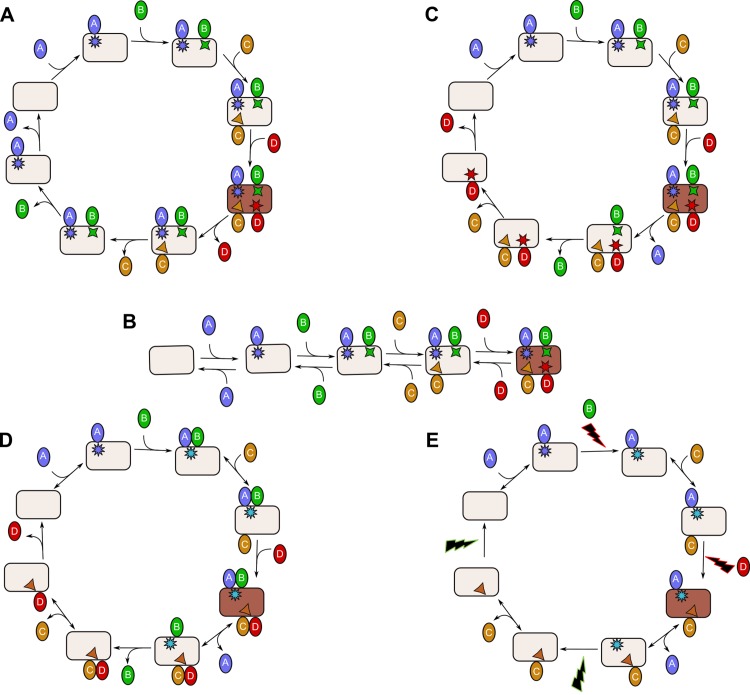
Cyclic mechanisms for transcription activation/inactivation, illustrated for four TFs. **A.** Cyclic mechanism with irreversible association and dissociation of the TFs, first-in, last-out. **B.** The linear mechanism identical to A. **C.** Feasible cyclic mechanism with sequential irreversible association and dissociation (first-in, last-out) of four TFs, each accompanied by the creation and later removal of a specific covalent modification of the chromatin. **D.** Batch-wise cyclic mechanism requiring only half the covalent chromatin modifications. **E.** Ticking mechanism, in which transient association of TFs suffices to tick the chromatin irreversibly.

An alternative to reversing on a linear scheme is a combination of a forward linear pathway and a separate reverse linear pathway; together constituting a cycle. In Fig 2A, four TFs bind irreversibly to and (a number of steps later) dissociate irreversibly from their DNA-binding site, referred to as a response element. The covalent histone modifications are shown by the various symbols in the rectangles. In the simplest irreversible cyclic mechanism (Fig 2A) the regulatory factors dissociate according to a last-in, first-out principle. In the S4 Text we show that this mechanism corresponds to the one Fig 2B and that this mechanism is too linear to be effective. A mechanism in which TFs dissociate irreversibly on the basis of a first-in, first-out principle (Fig 2C) is able to attain much higher transcriptional activity than the equilibrium binding model, whilst transition times between active and inactive gene states are also much shorter. Although every step in Fig 2C is irreversible, the cycle could also operate batch-wise (leading to a considerable advantage, see above): one or a few regulatory factors could bind reversibly after which one would bind really strongly (nearly irreversibly; so, the bound state has a long lifetime), thereby fixing the information that all regulatory factors have bound (see Fig 2D). A mechanism in which chromatin is ‘ticked’ or ‘stamped’ upon encountering a TF, which subsequently dissociates (Fig 2E), is equally competent kinetically. We call this the ‘ticking mechanism’. We note that the most realistic mechanism for leaving marks on the chromatin, following the activity of a particular short-lived protein complex, is via histone modifications, as we doubt that many regulatory proteins can stay bound to chromatin for tens of minutes without covalent interactions.

Our analysis thus far has used criteria of regulatory sensitivity and the speed of regulation of transcription to show that a ‘ticking’ transcription cycle mechanism is an attractive mechanism for eukaryotic genes that are regulated by multiple TFs. There is considerable experimental evidence that shows that this mechanism is indeed operative (S5 Text).

### Experimental evidence for the ticking mechanism

The ticking mechanism described above is consistent with the wealth of experimental evidence on the role of chromatin covalent-modification and remodelling in regulation of eukaryotic gene transcription [3, 5–7, 9, 10, 22, 44]. There are many examples of histone modification recruiting new modifiers, and subsequent modifications recruiting new proteins actively involved in transcription induction or cessation. For instance, during promoter activation, H4R3 methylation, mediated by the methylase PRMT1, increases the affinity of histone acetyltransferase p300 for chromatin [7]. Some acetylation marks, e.g. H3K14Ac, H4K16Ac, increase binding of chromatin remodelling complexes in various experimental systems [45, 46] while others (H3K9Ac, H4K12Ac, H4K8Ac) attract basal TFs required for initiation [47, 48]. This could potentially create an orderly sequence of modifications of a chromatin site thus providing the ticking mechanism of the gene activation process [22].

**Fig 3:**
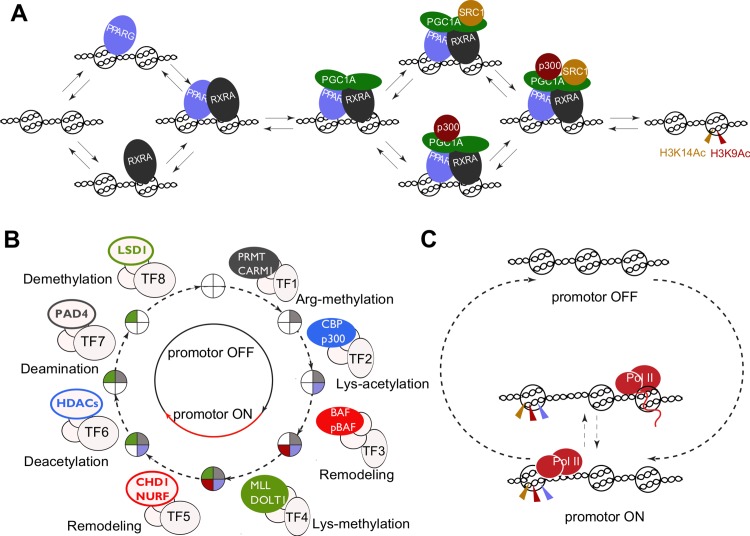
A ticking mechanism for gene switching between OFF and ON states. **A.** An example of a transition in gene (in)activation is given by the assembly of the protein complex composed of the two TFs, PPARγ and RXRα, the co-activator PGC1α and the histone acetyltransferases SRC1 and p300 [49]. The assembly follows a preferentially random mechanism and is followed by modifications of the histone H3 residues K3 and K9. **B.** The cycle of chromatin transitions associated with gene-expression switching in mammalian cells is show, this cycle is driven by covalent histone modifications and nucleosome remodelling. The proposed sequence was reconstructed from literature (see S5 Text). **C.** During the period of gene switching from the ON to OFF state, RNA polymerase II can bind to the transcription start site (TSS) and subsequently escape and initiate elongation. Given that the duration of the effective ON state is longer than RNA polymerase II binding and TSS escape, multiple polymerase molecules can leave the TSS per ON phase [20, 51, 52].

There is also ample evidence for TFs forming complexes with each other and with co-factors that have “ticking activity”. One example is the PPARγ-RXRα heterodimer that forms a complex with the co-activator PGC1α and the histone acetyltransferases CBP and SRC1 (Fig 3A) [49]. Another example is the repressive complex Mi-2/NurD that includes the chromatin remodeller Mi-2, the histone deacetylases 1 and 2, and the methyl CpG-binding protein MBD, which can also bind repressive TFs [50]. Fig 3B shows the possible transitions’ sequence between ‘ON’ and ‘OFF’ phases of the promoter cycle based on literature data (see S5 Text for full description and references). The ‘ON’ state is attained through three successive ticks, or irreversible modifications: arginine methylation, lysine acetylation and chromatin remodelling that make the transcription site accessible to basal TFs. This is the state during which RNA polymerases are assembled into transcriptionally competent complexes, and start the elongation of an mRNA transcript. The RNA polymerase II-associated complexes then add a fourth mark—methylation of a lysine group(s). The inactivation of the TF-binding site follows a similar mechanism of four successive protein complex formations, now accompanied by the removal of ticks from the chromatin. The transcriptionally active state of chromatin can in principle persist during de-ticking, depending on the order of the deactivating events. Each transition in the cycle could be dependent on DNA-binding TFs, which would regulate the transcription rate by changing the duration of ‘ON’ or ‘OFF’ states.

In the proposed mechanism, the sequence of chromatin modifications proceeds in a defined order. When the activation and inactivation routes would cross however, as would happen in a random mechanism, this would obviously disturb the progress marking, causing the system to erroneously move backward or forward, and skip steps. These considerations may explain the preference for a unique order of histone mark addition/removal and specificity of each partially assembled complex to a chromatin state.

### Precise transcription cycle times, despite inherent molecular noise, can cause transient transcriptional oscillations at the population level

The mean waiting time for a single TF-binding event equals its standard deviation, making its timing very imprecise. The time to complete a defined sequence of *n* identical first-order reactions follows an Erlang distribution [30]. The variance in the duration of such a sequence, denoted by ⟨*δ* ^2^ *τ*⟩, equals *n/k* ^*2*^. The noise in the duration of the sequence, defined as ⟨*δ* ^2^ *τ*⟩/⟨*τ*⟩^2^ equals *1/n*. Therefore, the noise equals 1 for the single step mechanism but it is much less for a sequence of such steps: a sequence of reactions with identical duration has a more precise completion time than any of its component reactions. This conclusion holds true if a sufficient number of the steps in the cycle have different but comparable durations, but falters should one reaction be much slower than all the others, then the noise increases.

**Fig 4:**
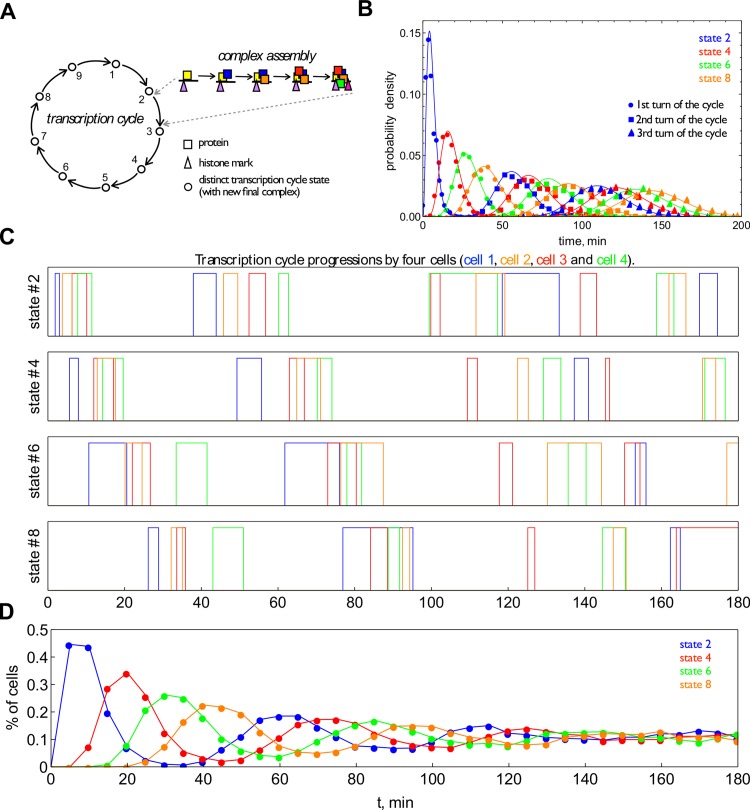
Precision of transcription cycle duration leads to transient population level transcription cycles. **A.** We consider a transcription cycle of 9 chromatin states. Each transition involves the formation of a single protein complex consisting of 5 proteins following a particular semi-random assembly mechanism followed by an irreversible histone modification event. **B.** Probabilities to observe states 2, 4, 6, and 8 in the population as function of time, obtained by modelling 2,500 individual cells that started in promoter state 1. **C.** Durations of the 4 chromatin states calculated for 4 cells from the population. **D.** Stochastic simulation of the transcriptional cycling performed for 2,000 gene copies. The probability of the presence of the four successive states was calculated as a function of time; colours correspond to the state as labelled in **B**.

We modelled a sequence of nine chromatin-state transitions as approximation of an entire transcription cycle. Each of the transitions involved the reversible assembly of a complex of five proteins followed by irreversible histone modification (Fig 4A). In total 45 regulatory proteins were involved. We considered a batch process, i.e. a preferentially random-assembly mechanism on chromatin for protein complex formation, with a strictly ordered sequence of the chromatin modifications guiding the sequence of complex assemblies and sensitization of the chromatin for the assembly of the next protein complex. We chose realistic values for the rate constants and protein abundances (see S3 Text and S3–S5 Tables). Fig 4C shows cycling through several promoter states by four individual cells after transcription was activated in each of them at the same time. We observe that, for example, state 2 occurs in the first cycle in all cells almost simultaneously; and in subsequent cycles the occurrence of this state slowly loses synchrony across the four cells, as indicated by the increasing dispersion of the occurrence time. This means that the cells are progressing through the transcription cycle slowly (as compared to the TF binding time scale) and synchronously in the beginning, but desynchronize over time. This can be further illustrated by plotting the probability of observing each of the four states in a population as function of time (Fig 4B).

To illustrate how the stochasticity of assembly times leads to transient de-synchronization of a population of cells, we will consider what occurs with increasing number of cycles of n steps. The overall waiting time distribution to complete *N* ^*th*^ cycle becomes narrower with an increasing number (*N*) of cycles: $\frac{\langle \delta^{2}\tau_{N}\rangle }{\langle \tau_{N}{}^{2}\rangle }={\left(n\cdot N\right)}^{-1}$. To quantify the effect of de-synchronization, we consider the noise in the timing of the end of the *N*-th cycle relative to the mean duration of a single cycle, i.e. using $\langle \tau_{N}\rangle =N\langle \tau \rangle :\frac{\langle \delta^{2}\tau_{N}\rangle }{{\langle \tau \rangle }^{2}}=\frac{N}{n}$. This relation shows that cells will progressively become asynchronous with increasing number of completed transcription cycles, and that phase-noise in the *N* ^*th*^ cycling time increases linearly with *N*. At the same time, it shows that more transitions per transcription cycle, *n*, tend to prolong the persistence of population level synchrony. These relations explain the behaviour calculated in Fig 4.

We simulated the stochastic dynamics for a population of 1,000 cells that had simultaneously started transcription activation of two gene copies. Fig 4D shows the fractions of cells that are in a given state at any moment in time (also shown for a single cell in Fig 4C).

**Fig 5:**
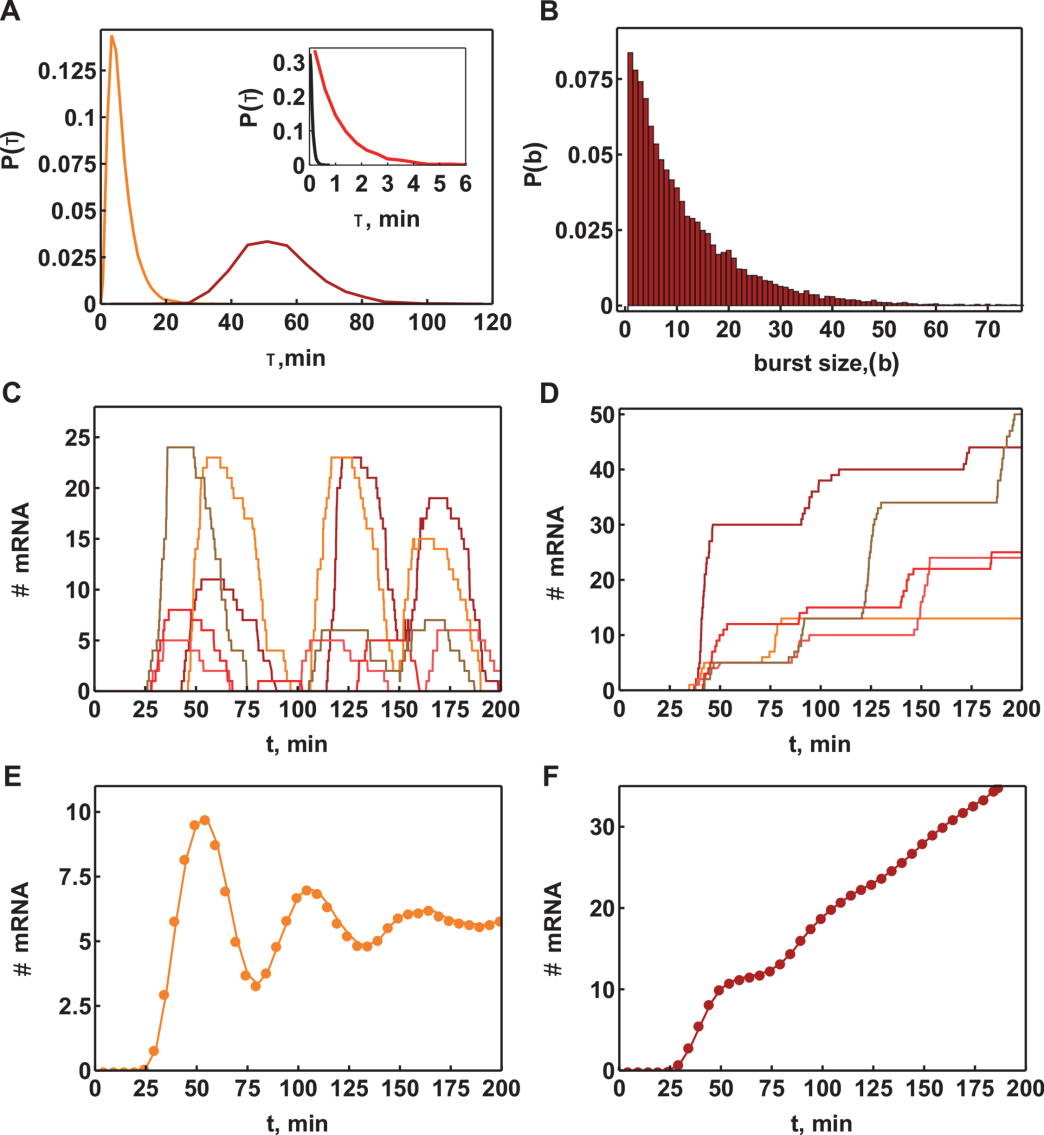
Stochastic dynamics of mRNA production in the 9-state transcription ratchet model. **A.** Overall cycle time distribution function P_CYCLE_(τ) with an average time of 54 min (dark red line; as produced by the ratchet model for transcription activation) and the time distribution of the chromatin state permissive to polymerase binding with an average of 6 min (orange line). Inset: waiting time distribution function P_ON_(τ) for the ON state (red line); and for regulatory region escape event P_ESCAPE_(τ) (black line), which have an average of approximately 1 and 0.1 min and are exponentially distributed, respectively. **B**. Distribution function of burst sizes p(b) produced by the model with a calculated average of ~11 molecules per burst. **C**. Due to the fairly precise waiting distribution of the cycling times, five cells that are activated simultaneously will display synchrony in mRNA production for some time. **D**. A similar picture is observed in the case of lack of degradation where bursts result in a step-wise increase of mRNA. **E**. Population level response of mRNA in the presence of mRNA degradation. **F**. Population level response of mRNA in the absence of mRNA degradation.

Fig 4D shows that population level transcriptional cycles are observable for around 100 min and decrease in amplitude over time due to de-synchronization as expected. The transcriptional cycling time that we predict with this realistically parameterized model is approximately 54 min. The predicted waiting time distribution of the cycling time indeed peaks (Fig 5A), as expected for a multi-step sequential process.

### Bursts in the system

The number of RNA polymerase molecules that initiate elongation per transcription cycle depends on the tendency of genes to engage in a phenomenon called re-initiation [20, 51–53]. Many experimental studies have shown that mRNA is produced in bursts [14, 54–56] of variable size [57, 58]. We incorporated RNA polymerase II binding and promoter escape during the permissive ON state into the model. We considered promoter state 5 to be transcriptionally permissive. The overall lifetime of the state is around 6 min (Fig 5A, orange line). However, as we assumed competition between polymerase and deactivating complex, the effective permissive state only occurs while the deactivating complex is not bound and its lifetime is close to 1 min (Fig 5A inset, red line). The polymerase concentration in the model was taken higher than the concentrations of other TFs, estimate supported by the experimental copy number data (S2 Fig). Therefore, polymerase binding and promoter escape time had an average of 0.1 min (Fig 5A inset, black line), a much shorter time than the lifetime of the effective ON state, resulting in multiple transcripts being initiated during most of the permissive periods. The average burst size should then be 10 mRNAs/cycle and the distribution of burst size can be shown to be geometric; which is indeed the case for calculated burst size distributions in Fig 5B. This finding is in good agreement with experimental observations that mRNA burst-size distributions for regulated genes across a cell population are often geometric [54, 55]. The contribution of the stochasticity of transcriptional bursts, as generated by the ratchet mechanism, to the total stochasticity of transcript concentrations in a cell depends also on factors, independent of the ratchet mechanism [59].

Whether bursting will occur in synchrony across cells depends on whether the cells were induced at the same time. Fig 5C and 5D show the prediction of mRNA trajectories in five individual cells for the 9-state ratchet cycle model that were induced at the same time (Fig 4A). The model predicted mRNA bursting: all five modelled cells fired mRNAs periodically and for the first 2 h they did this in fair synchrony. When mRNA decay was rapid (Fig 5C), most of the mRNA was degraded during the promoter OFF period. For multiple cells, this led to the prediction of population level transient oscillation in mRNA levels (Fig 5E). If no mRNA degradation occurred on the considered timescale the bursts led to step-wise accumulation of mRNA in individual cells (Fig 5D) as well as on the cell population level (Fig 5F).

### A transcription (in)activation cycle model with realistic kinetics can reproduce experimentally observed population-level transcriptional cycling

The model developed in the previous sections explains a number of experimental observations dealing with cyclical changes in protein occupancy, nucleosome modifications, looping of eukaryotic regulatory regions, and transcriptional bursts. The transcriptional cycling periods that have been observed experimentally vary between 40 and 90 min [24, 25, 27, 28, 60], in line with the above predictions for the 9-state ratchet cycle model.

**Fig 6:**
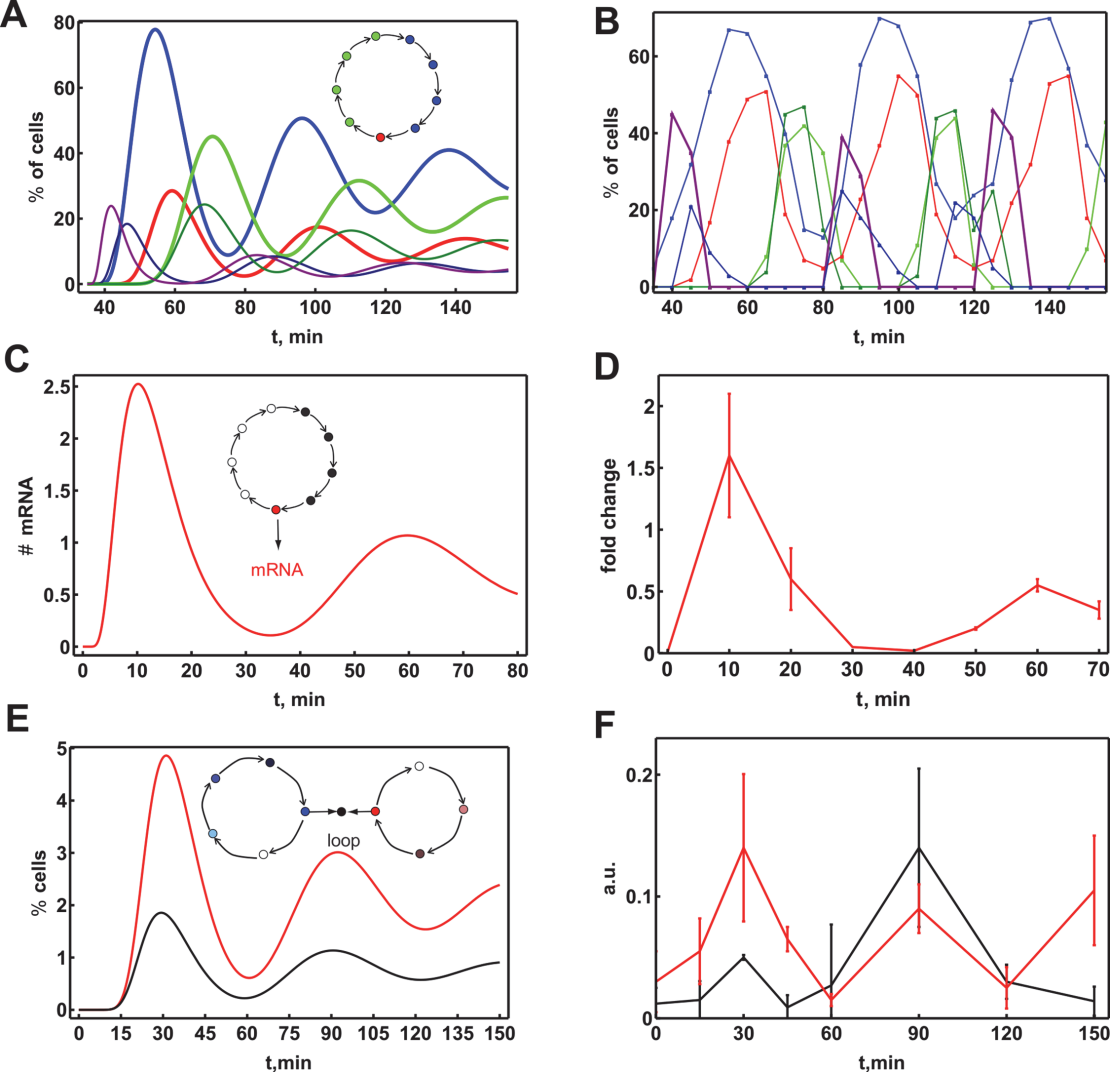
The transcription clock model can explain existing experimental data. The single regulatory region model with nine chromatin transitions (presented in Fig 4) with adjusted protein concentrations can qualitatively reproduce the ordered cyclic co-regulator binding as observed in various experiments reported in literature. **A.** Time courses of the average regulatory region occupancy on the cell population level. The plotted regulatory region occupancy states were chosen to fit the data profiles (see S6 Table). Inset shows the schematic representation of the cycle of chromatin states, colours correspond to plotted states. **B.** Experimental data, simulated in panel A, reproduced from [27]: blue: the TF estrogen receptor, red: RNA polymerase II, purple: co-regulator CARM1, dark blue: histone acetyltransferase CBP, green: HDAC1, dark green: chromatin remodelling factor Brg1. **C.** The same model, with corrected protein concentrations to match cycle duration, extended with mRNA synthesis and degradation, reproduces population level mRNA cycling as observed experimentally and shown in: **D**. Data reproduced from [24]. **E.** Simulation of the model modified to include the TSS and a RE, which form a loop during the initiation phase (red complex). This model can reproduce looping (black line) and polymerase-binding data (red line) as observed experimentally for the regulatory region of the *CDKN1A* gene by [28]; experimental data shown in **F**. The arbitrary units of regulatory region looping data (between TSS and RE 3) are re-scaled by a factor of 10,000 for ease of visualization. The parameter adjustments are documented in the S4 and S5 Tables.

The most detailed dataset available is for the regulatory region of the estrogen-responsive trefoil factor 1 (*TFF1*) gene [27]. It exhibits the orderliness of the binding events that is a component of cyclic ratchet model: first, co-activator complexes containing histone acetyltransferases and histone methyltransferases bind, then the basal TFs and RNA polymerase II, and finally the de-activator complexes, containing remodelling/HDAC activity. This experimental data (Fig 6B) can be reproduced fairly well by a 9-state cycle model with realistic kinetic parameters (Fig 6A). The de-synchronization observed in the experiment was however slower than in the model simulations. This could be explained by an even higher number of proteins and chromatin modifications involved serially in the initiation process than currently known experimentally, by more peaked waiting time distributions for individual cycle transitions, which could again be due to multi-step serial processes, or by differences in kinetic parameters. A clear example where the de-synchronization predicted by our model is observed experimentally has been provided by the measurement of the TF occupancy on chromatin, using fluorescence-based methods in single cells [24].

Population level oscillations in mRNA concentrations as predicted by our cycle model (Fig 5E) have also been observed experimentally (Fig 6D). The yeast metallothionein (*CUP1*) gene, displayed 50-min transcriptional cycles of its main TF, Ace1p, upon activation and the *CUP1* mRNA was oscillating at the same frequency [24]. Adjustment of the protein concentrations and the consideration of two promoter states (PR2 and PR3), which can each be transcriptionally permissive, allows for qualitative correspondence of the model simulations and experimental data.

Evidence for reversible looping between a response element and the TSS has been found in the regulatory region of the human cyclin-dependent kinase inhibitor 1A (*CDKN1A*) gene, which is regulated by several REs for the TF VDR [28]. Some of the co-regulators were shown to oscillate at the population level with a 60-min period. The experimental data (Fig 6F) can be reproduced by our model (Fig 6E) if the looping event between the distant RE and TSS in the model occurs during the chromatin state corresponding to RNA polymerase II binding.

## Discussion

In this study, we asked how transcription regulation of conditionally active genes in eukaryotes could be organized so that it is both fast enough, given diffusion limitations, and sensitive enough towards a large number of TFs. We showed that neither an equilibrium binding mechanism nor a more irreversible binding mechanism, reversing its steps upon inactivation, could be fast and sensitive enough. We then showed that the next simplest, competent mechanism is a batch-like multi-step process of transcription activation, followed by a separate batch-like multi-step inactivation process, together constituting a transcription (in)activation cycle. Each step would correspond to the reversible association of a limited number of TFs leading to irreversible marking of the transcription activation complex, after which the TFs dissociate. This transcription (in)activation cycle should not be confused with the more restricted use of the term transcription cycle when it refers to the process of an RNA polymerase producing an entire mRNA. We have a much more involved process of gene activation and transcription initiation in mind.

If one were to specify that histone modification is the process that marks the completion of a step in the multi-step process and sensitizes the local chromatin region for the assembly of the next protein complex, this model comes close to the accepted view of transcription activation in eukaryotes [7, 10, 22, 44]. The difference is that we derived this view as a requirement for delivering kinetic competence, revertibility and sensitivity, whereas the accepted view is based on experimental observations of the transcription activation process. All in all, our study suggests that we have found a plausible explanation as to why transcription (in)activation of many regulated genes in eukaryotes is organized the way it is, and why it is different from the activation of transcription that is regulated by a small number of TFs, such as in prokaryotes, or the house-keeping genes.

Metivier *et al*. [27] proposed a branched mechanism for the transcription cycle at the *TFF1* promoter. Our model can straightforwardly be extended with such details. We considered such an extension in a minimalist manner when we incorporated the transcription re-initiation mechanism to allow for variable burst size of the transcription cycle.

In our model, a sequence of protein complex assemblies intermitted by covalent-modification of histones to mark the phase of the transcription cycle, leads to a fairly deterministic duration of the active transcription state. This has two consequences. Firstly, our model explains how the duration of the entire transcription cycle could be close to deterministic, i.e. clock-like. Secondly, in the transcription re-initiation formulation of the model the number of transcription (re-)initiations per cycle becomes more deterministic. This means that the eukaryotic transcription cycle can be both clock-like in terms of duration and quantal in terms of its activity. This is within the limit of many molecular events in series with similar reaction rates. To what extent real genes function within this limit is unclear. Recent experiments indicate that the ON and OFF durations of genes can have non-exponential waiting time distributions, which is in agreement with our predictions [58, 61, 62]. The clock-like nature of the model underlies the tendency of cells in a population to display transiently synchronous transcription activity upon simultaneous activation from the same initial state. The model does not, however, exclude a possibility of the transcription cycle times being less precise, due, for instance, to the presence of a very slow step in one or more of the cycle transitions, or a possibility of variable gene induction times due to heterogeneity of the initial promoter states in a cell population.

We have shown that our model explains precise durations of the entire transcription cycle of about 1 hour on the basis of molecular processes that are faster than 1 per minute. This is because of a sequence of several multi-step processes, each of which is consolidated by irreversible marking (ticking). This ticking corresponds to a ratchet mechanism, but the overall process corresponds to a clock mechanism as already proposed by Reid *et al*. [22]. A mechanical clock has the same type of ratchet mechanism and is similarly accurate at longer time scales: the timing of its individual ticks is not accurate, but the timing of large numbers (for example 60) of its ticks is. From a coarse-grained perspective, the transcription activation cycle model assumes that eukaryotic genes switch between transcription active ON states and ditto, inactive OFF states. The duration of these phases could be under the control of some regulator [56, 63, 64]. The same applies to burst size. From this perspective, transcription rates can be controlled in different ways. A suitable definition of transcription rate would be the mean burst size divided by the mean cycle time: $\frac{\langle b\rangle }{\langle \tau_{ON}\rangle +\langle \tau_{OFF}\rangle }$. Control of transcription rate can then be achieved via modulation of burst frequency (cycle duration; the FM mode) or burst size (the AM mode). Skupsky *et al*. [65] found evidence for regulation of burst size rather than frequency. The transcription cycle models we presented can accommodate both mechanisms.

Not all protein complexes involved at the various stages of the transcription cycle assemble on the chromatin, some may already be assembled in the nucleoplasm, such as mediator complex [66]. These pre-assembled complexes do not affect the total duration of the transcription cycle and its precision, as they bind in one step. However, even if all protein complexes would assemble in the nucleoplasm, which we know is not the case, histone marking would be still advantageous to make sure that they bind in the correct order.

In our model we propose that histone modification function as marks of the progress of the transcription cycle, because they have a longer lifetime than protein complexes—proteins reside on chromatin only for several tens of seconds. This does not mean that all histone marks should be remembered along the entire transcription cycle. We expect the minimal life of a histone mark to be related to the time that it takes to form the next-in-line protein complex. So, if protein complex *i* has been formed after which mark i is added, the lifetime of mark *i* should be long enough to make sure that the protein complex *i+1* has had its time to form and leave mark *i+1*. Since marks can always be removed by accident by an enzyme, or fall off spontaneously, it makes sense that a short memory of previous marks should be present on chromatin, say in addition to mark i, mark *i-1* and *i-2*, to make sure that if mark *i* gets removed by accident the transcription process does not erroneously reset to its resting state. Considering that the formation of a protein complex on chromatin takes several minutes, we would therefore expect that histone modifications that mark the progress of the transcription cycle stay for about 10 minutes or more on chromatin.

Transcription dynamics differ between prokaryotes and eukaryotes [14, 19–21, 54, 55, 67–69]. Eukaryotes tend to display transcription bursts, which in prokaryotes have only been found under conditions of leaky transcription repression [56, 64, 70], and occur infrequently across the majority of genes in *Escherichia coli* [71]; in contrast to what is found in *Saccharomyces cerevisiae* [72, 73]. RNA polymerase II assembly, and its escape from the regulatory region, only take place during a fraction of the entire transcription cycle, suggesting that many eukaryotic genes are prone to transcriptional bursting even under conditions of high transcriptional activity [24, 27, 54, 55]. The mechanisms for transcriptional bursts in bacteria [64, 74, 75] are likely very different in molecular detail from the mechanisms in eukaryotes, even though they can be coarse-grained to a similar mechanism giving rise to a variable number of RNA polymerases that initiate transcription during a single ‘ON’ state of the gene [59].

Transcriptional cycling differs from regular oscillations induced by a negative feedback loop, as found, for example, in NADH fluorescence and glycolytic activity in yeast [76], the dynamics of calcium concentrations [77], and the activities of NF-κB [78] or the kinase ERK [79]. In the latter examples the dynamics of metabolite or signal transduction factor pools are coupled through nonlinear kinetics. In some of these cases sustained oscillations have been observed, and for one of these the requisite active synchronization mechanism requiring dynamic communication between individual cells, has been elucidated [80]. The mechanism we propose for the transient oscillations at the population level is entirely cell-autonomous, i.e. no active communication between cells is involved. For transcriptional cycling, we propose that the transient cyclic dynamics at the population level are the consequence of a simultaneous start and accurate durations.

## Models

The mass balances, rate equations and parameters for protein-assembly and complete transcription initiation models were generated by using custom algorithms implemented in Mathematica 7.0. ODE system simulation were done using NDSolve function; stochastic simulations—by implementing direct-method of Gillespie algorithm [81]. For detailed description of the models’ structures, parameters and initial conditions see S3 Text, S1–S6 Tables and S6 Fig. All Mathematica files are available as part of Supporting Information (S1 Dataset).

## Supporting Information

S1 FigEffect of TF interaction coefficient on the sensitivity of the transcription rate to a single TF concentration.The value of the interaction coefficient, *β*, that minimizes (*Φ*(***T***, *n*, *β*)−*ϕ*(*T*))^2^ for a range of values for the TF concentration (*T*; from 0 to 20-times the affinity constant, *K*) is plotted as function of the number of TFs, n. The inset shows the dependency of the squared difference between *Φ*(***T***, *n*, *β*) and *ϕ*(*T*) as function of *β* and shows that a minimal value for *β*, the optimal value, exists for each value of *n* (ranging from 2 to 20). The optimal value of *β* is defined as $\beta_{opt}=argmin\left[\sum_{i=0}^{40}{\left(\Phi \left(i\Delta \alpha ,n,\beta_{opt}\right)-\phi \left(i\Delta \alpha \right)\right)}^{2}\right]$ with Δα = 0.5; hence, *α* = *T* / *K* ranges from 0 to 20. This notation works as follows: “argmin[*f(λ)*]” returns the value of variable λ that minimizes the function f(λ).(PDF)

S2 Fig Copy number distributions found experimentally for different types of proteins involved in eukaryotic transcription. Distribution of copy numbers were calculated for sequence specific TFs (blue), histone modifiers (green) and general TFs (red) based on the proteomic data from two mouse cell(line)s (Azimifar *et al*. [38] and Schwanhausser *et al*. [41] and two human cell lines (Beck *et al* [39]. and Nagaraj *et al*. [40] Calculated means of distributions are indicated by vertical lines. Lists of proteins with specific functions (sequence specific TFs, histone modifiers, GTFs), were created based on relevant GO-terms and further curated to ensure that only proteins for which experimental evidence for their functions is provided were included. The corresponding UniProt IDs lists were checked against the corresponding ID lists provided in the proteomic measurement publications and copy number measurements for proteins found used to calculate the distributions. The curated lists of Gene Names/UniProt IDs used as well as the lists of all copy numbers used for the distribution calculations are provided as part of Supporting Information (Modeling files.zip).
(PDF)

S3 FigAssembly time of protein complexes depending on the mechanism.The protein complex assembly time as function of the dimensionless dissociation constant of trimer formation for assembly mechanisms that differ in organization. In addition to the ordered irreversible assembly mechanism, four reversible mechanisms are considered for the assembly of a trimeric complex on chromatin as indicated by the diagrams on the right. They are compared to the irreversible ordered-sequential mechanism illustrated on top. The tendency to fall apart is captured by the apparent dissociation constant $K_{D}^{\prime }$. The inset gives the probability distribution of assembly times for the sequential-ordered reversible mechanism (at an apparent *K*_*D*_ = 0.1). The red dot gives the mean assembly time.(PDF)

S4 Fig Mechanisms of protein complex assembly and their duration depending on the sequence and association rate constant. **A.** All complexes that can be formed in the *N* = 5 random mechanism (both in RE and in cytoplasm) that is used in the models are presented; the complexes that can bind RE are shown as bound forms. The lists of complexes that are used in preferentially random and sequential assembly mechanism are given in S2 Table. **B**. Half-time of promoter modification for a range of *k*_*on*_^*eff*^ values (product of protein concentrations and their *k*_*on*_) for nine different protein assembly mechanisms (sequential—Seq; preferentially random—PR; random—Ran; on chromatin—Chr; nucleoplasmic—Nuc) involving 5 proteins as indicated. In all instances *K*_*eq*_ = 10^9^ M (here defined as k_on_/k_off_; k_off_ is variable depending on the value of *k*_*on*_^*eff*^). The rate constant for the final irreversible acetylation of lysine 9 and lysine 14 *k*_*mod*_ = 60 min^-1^, i.e. much faster than the dissociation constant. **C**. Fraction of promoter binding at equilibrium as function of the *k*_*on*_^*eff*^ for the same set of mechanisms. In all simulations, the RE became ready for complex assembly at time zero. All models were simulated so as to be at equilibrium prior to time zero. At time zero the RE becomes available for binding.
(PDF)

S5 Fig Various mechanisms for linear transcription mechanism by 10 TFs, with their efficacy. **A**. Inactive (unless mass action irreversible) but then irrevertible; **B**. active, but irrevertible; **C.** active, forward hesitant (unless mass action irreversible) but then irrevertible, because of hesitation on the reverse route; **D.** Active, forward hesitation removed, but again irrevertible; **E**. Active, revertible; but with re-emerging hesitation problem.
(PDF)

S6 Fig Structures of detailed initiation models. Chromatin state transition scheme for the models presented—a basic 9-state promoter model (also used for simulating the Metivier *et al*. and Karpova *et al*. data) (A), a modified model with looping between distant RE and TSS (B) used to simulate the Saramäki *et al*. data. The chromatin states in which RNA polymerase II binding/mRNA production occurs are marked in colour. In scheme A, PR5 is considered in ON state for generic 9-state chromatin model, while for simulating Karpova *et al*. PR1+PR2 are considered permissive. Specific chromatin states plotted in Figs 4 and 6 in the main text are described in S6 Table. In every model a single chromatin transition corresponds to formation of an N = 5 protein complex on the PR/RE by preferentially random mechanism followed by a chromatin modification step (**C**) or an N = 4 protein complex on TSS likewise followed by a chromatin modification step (**E**). For simulating Karpova *et al*. complexes formed on PR2 contain polymerase and lead to transition to PR3 (**D**); likewise, PR3 can be bound to the same RNA polymerase II containing complex and its own de-activation complex. Each successful formation of an RNA polymerase II complex leads to the start of elongation. In model **B** (simulation of Saramäki *et al*.) the loop is formed between partially bound RE3 and RNA polymerase II bound to TSS3, leading to modification of polymerase that is ready for elongation (**F**). In all models elongation is modeled as a 30-step process that leads to formation of mature mRNA and release of the RNA polymerase II.
(PDF)

S1 Table Interaction matrix of protein complexes. A realistic interaction scheme for a complex of five proteins is presented in the form of an interaction matrix. The interaction matrix was used to produce the mass balance and rate vectors for the random assembly model.
(PDF)

S2 Table Complexes formed in preferential random and sequential assembly mechanisms. A customary algorithm was used to remove reactions involving complexes formed only in fully random mechanisms.
(PDF)

S3 Table Example of parameter calculation. A customary algorithm was used to calculate rate constants for the corresponding protein association and dissociation reactions.
(PDF)

S4 Table Parameters for transcription in 9-state promoter model. Protein association and dissociation rate constants and concentrations were calculated in the same way as shown in S3 Table. The modification rate constants were chosen in a way to provide the same effective rates as a single protein-binding step to reduce the noise in waiting times. The values are in the range for k_cat_ of chromatin modifying enzymes measured *in vitro* [s35-37]. Elongation and export times were estimated from data available in literature [s38-41] and modeled as a multi-step process (N = 30). A 30 min life-time was assumed; degradation was modeled as multi-step process with (N = 20). For models used to simulate data, additional parameters as well as parameters different from the main model are given. For simulation of the Karpova *et al*. data, the initiation rate constant was taken to be faster than the RNA polymerase II off-rate. The rates of mRNA elongation (modeled as N = 30 process) and degradation (modeled as N = 20 process) were adjusted to fit the data.
(PDF)

S5 Table Parameters for the promoter model with looping between RE and TSS for simulating data from Saramäki *et al*. Protein association and dissociation rate constants and concentrations were calculated in the same way as shown in S3 Table. Only the new mRNA synthesis was modelled. As the number of states is lower in this model, a lower degree of reversibility is required to reach comparable passive synchronization as in the 9-state model, hence the lower off rates. The on-rate of loop formation was taken as an estimation from data presented by Polikanov *et al*. (2007) [s42], which suggests that the average rate for DNA loop formation of comparable length *in vitro* is at least faster than 60 seconds. The loop was assumed to be stable (K_d_ = 10^12^) due to a big protein interaction surface. The polymerase modification step and promoter step were modelled explicitly. The protein concentrations were adjusted to fit experimental data.
(PDF)

S6 Table Summary of information on different promoter models. Specifications (structures and parameters) of each model are described, and the model states plotted in the main text figures are listed.
(PDF)

S1 Text Promoter sensitivity to multiple TFs in the equilibrium binding model. (PDF)

S2 Text Mean time of protein complex assembly reversible versus irreversible mechanisms. (PDF)

S3 Text Timing of the protein assembly mechanisms. (PDF)

S4 Text Revertible linear mechanism of transcription (in)activation. (PDF)

S5 Text Evidence for chromatin modification-driven promoter cycling mechanism. (PDF)

S6 Text Supporting Information references. (PDF)

S1 Dataset Modeling files. (ZIP)
