# Supplementary material for: Multiplex Eukaryotic Transcription (In)activation: Timing, Bursting and Cycling of a Ratchet Clock Mechanism
Source: PLoS Comput Biol. 2015 Apr 24;11(4):e1004236. doi: 10.1371/journal.pcbi.1004236 (PMC4409292; doi:10.1371/journal.pcbi.1004236)
Supplement: S1 Fig — The value of the interaction coefficient, β, that minimizes (Φ(T, n, β)−ϕ(T))2 for a range of values for the TF concentration (T; from 0 to 20-times the affinity constant, K) is plotted as function of the number of TFs, n. The inset shows the dependency of the squared difference between Φ(T, n, β) and ϕ(T) as function of β and shows that a minimal value for β, the optimal value, exists for each value of n (ranging from 2 to 20). The optimal value of β is defined as βopt=argmin[∑i=040(Φ(iΔα,n,βopt)−ϕ(iΔα))2] with Δα = 0.5; hence, α = T / K ranges from 0 to 20. This notation works as follows: “argmin[f(λ)]” returns the value of variable λ that minimizes the function f(λ). (PDF) [file pcbi.1004236.s001.pdf]

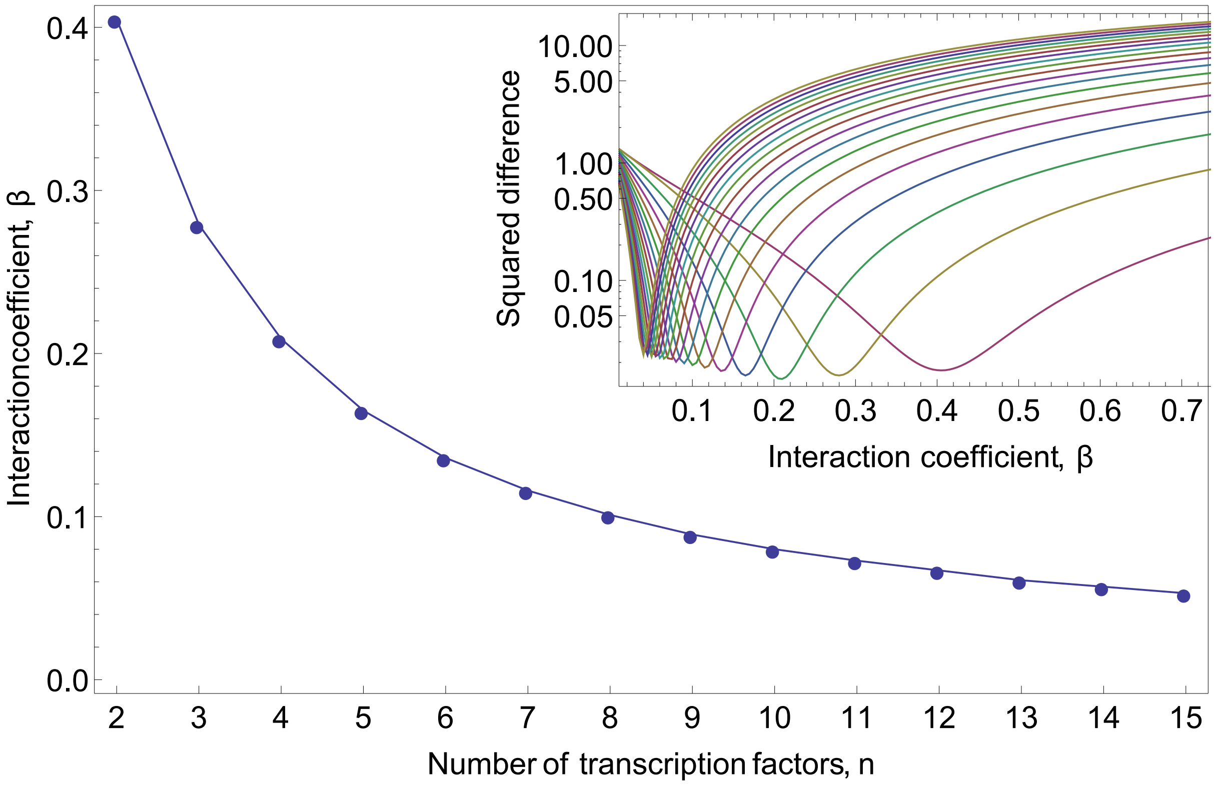

S1 Fig: Effect of TF interaction coefficient on the sensitivity of the transcription rate to a single TF concentration. The value of the interaction coefficient, β, that minimizes $(\Phi(\boldsymbol{T}, n, \beta) - \phi(T))^2$ for a range of values for the TF concentration (*T*; from 0 to 20-times the affinity constant, *K*) is plotted as function of the number of TFs, n. The inset shows the dependency of the squared difference between $\Phi(\boldsymbol{T}, n, \beta)$ and $\phi(T)$ as function of β and shows that a minimal value for β, the optimal value, exists for each value of *n* (ranging from 2 to 20). The optimal value of β is defined as $\beta_{opt} = argmin\left[\sum_{i=0}^{40}(\Phi(i\Delta\alpha, n, \beta_{opt}) - \phi(i\Delta\alpha))^2\right]$ with Δα=0.5; hence, $\alpha = T/K$ ranges from 0 to 20. This notation works as follows: "argmin[*f(λ)*]" returns the value of variable λ that minimizes the function f(λ).
