# Supplementary material for: Multiplex Eukaryotic Transcription (In)activation: Timing, Bursting and Cycling of a Ratchet Clock Mechanism
Source: PLoS Comput Biol. 2015 Apr 24;11(4):e1004236. doi: 10.1371/journal.pcbi.1004236 (PMC4409292; doi:10.1371/journal.pcbi.1004236)
Supplement: S2 Fig — Distribution of copy numbers were calculated for sequence specific TFs (blue), histone modifiers (green) and general TFs (red) based on the proteomic data from two mouse cell(line)s (Azimifar et al. [38] and Schwanhausser et al. [41] and two human cell lines (Beck et al [39]. and Nagaraj et al. [40] Calculated means of distributions are indicated by vertical lines. Lists of proteins with specific functions (sequence specific TFs, histone modifiers, GTFs), were created based on relevant GO-terms and further curated to ensure that only proteins for which experimental evidence for their functions is provided were included. The corresponding UniProt IDs lists were checked against the corresponding ID lists provided in the proteomic measurement publications and copy number measurements for proteins found used to calculate the distributions. The curated lists of Gene Names/UniProt IDs used as well as the lists of all copy numbers used for the distribution calculations are provided as part of Supporting Information (Modeling files.zip). (PDF) [file pcbi.1004236.s002.pdf]

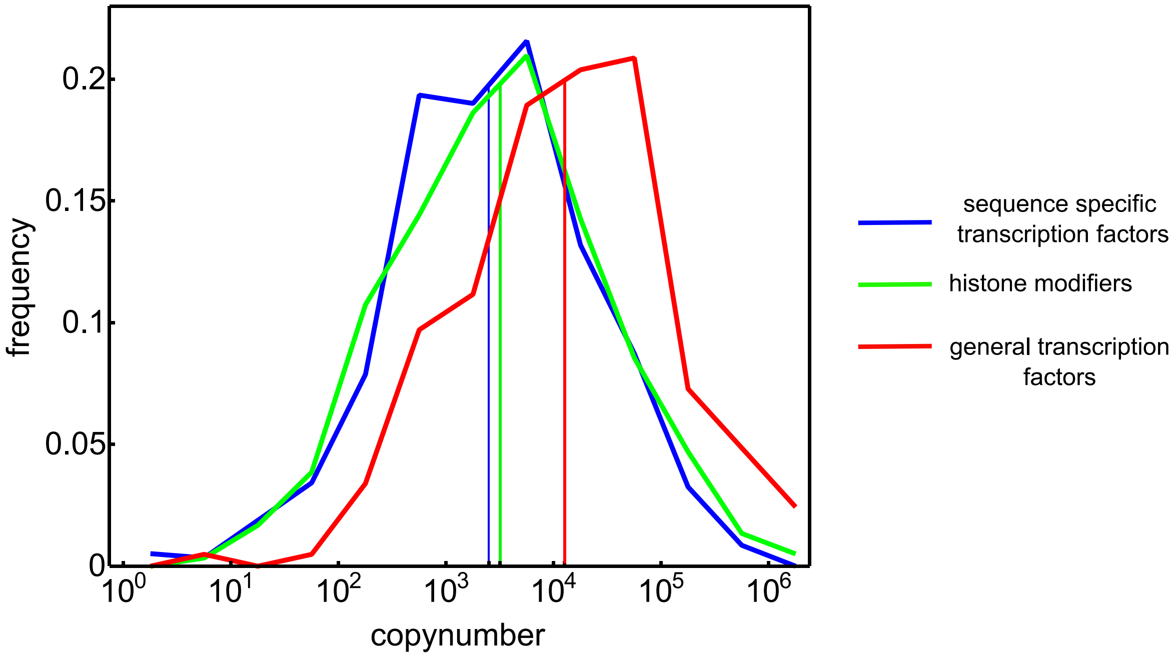

S2 Fig: Copy number distributions found experimentally for different types of proteins involved in eukaryotic transcription. Distribution of copy numbers were calculated for sequence specific TFs (blue), histone modifiers (green) and general transcription factors (red) based on the proteomic data from two mouse cell(line)s (Azimifar *et al,* Cell Metabolism, 2014 and Schwanhausser *et al,* Nature, 2011) and two human cell lines (Beck *et al,* MSB, 2011 and Nagaraj *et al,* MSB, 2011). Calculated means of distributions are indicated by vertical lines. Lists of proteins with specific functions (sequence specific TFs, histone modifiers, GTFs), were created based on relevant GO-terms and further curated to ensure that only proteins for which experimental evidence for their functions is provided were included. The corresponding UniProt IDs lists were checked against the corresponding ID lists provided in the proteomic measurement publications and copy number measurements for proteins found used to calculate the distributions. The curated lists of Gene Names/UniProt IDs used as well as the lists of all copy numbers used for the distribution calculations are provided as part of Supporting Information (Modeling files.zip).
