# Supplementary material for: Multiplex Eukaryotic Transcription (In)activation: Timing, Bursting and Cycling of a Ratchet Clock Mechanism
Source: PLoS Comput Biol. 2015 Apr 24;11(4):e1004236. doi: 10.1371/journal.pcbi.1004236 (PMC4409292; doi:10.1371/journal.pcbi.1004236)
Supplement: S3 Fig — The protein complex assembly time as function of the dimensionless dissociation constant of trimer formation for assembly mechanisms that differ in organization. In addition to the ordered irreversible assembly mechanism, four reversible mechanisms are considered for the assembly of a trimeric complex on chromatin as indicated by the diagrams on the right. They are compared to the irreversible ordered-sequential mechanism illustrated on top. The tendency to fall apart is captured by the apparent dissociation constant KD′. The inset gives the probability distribution of assembly times for the sequential-ordered reversible mechanism (at an apparent K D = 0.1). The red dot gives the mean assembly time. (PDF) [file pcbi.1004236.s003.pdf]

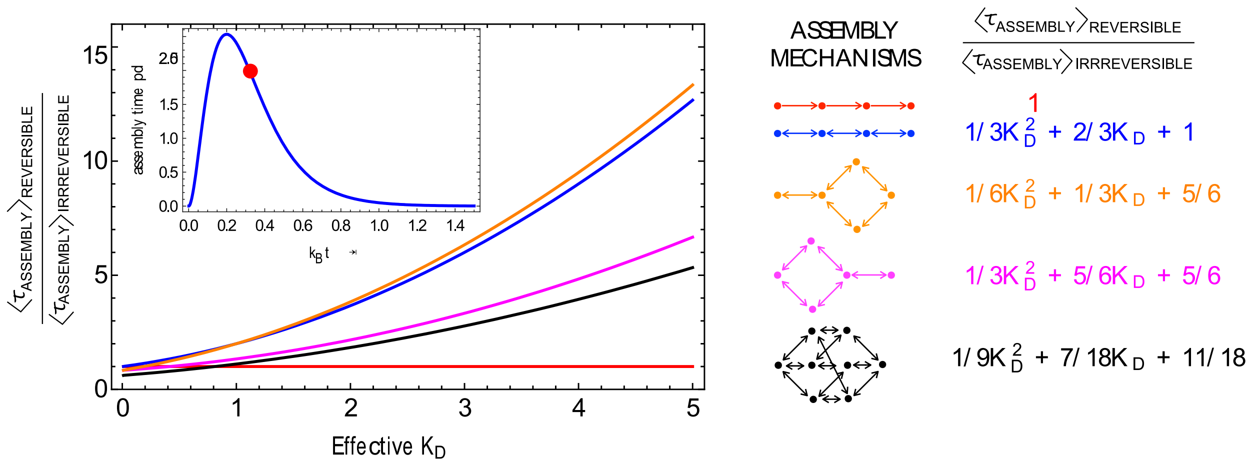

S3 Fig: Assembly time of protein complexes depending on the mechanism. The protein complex assembly time as function of the dimensionless dissociation constant of trimer formation for assembly mechanisms that differ in organization. In addition to the ordered irreversible assembly mechanism, four reversible mechanisms are considered for the assembly of a trimeric complex on chromatin as indicated by the diagrams on the right. They are compared to the irreversible ordered-sequential mechanism illustrated on top. The tendency to fall apart is captured by the apparent dissociation constant $K_D^{\prime}$. The inset gives the probability distribution of assembly times for the sequential-ordered reversible mechanism (at an apparent $K_D = 0.1$). The red dot gives the mean assembly time.
