# Supplementary material for: Multiplex Eukaryotic Transcription (In)activation: Timing, Bursting and Cycling of a Ratchet Clock Mechanism
Source: PLoS Comput Biol. 2015 Apr 24;11(4):e1004236. doi: 10.1371/journal.pcbi.1004236 (PMC4409292; doi:10.1371/journal.pcbi.1004236)
Supplement: S4 Fig — A. All complexes that can be formed in the N = 5 random mechanism (both in RE and in cytoplasm) that is used in the models are presented; the complexes that can bind RE are shown as bound forms. The lists of complexes that are used in preferentially random and sequential assembly mechanism are given in S2 Table. B. Half-time of promoter modification for a range of k on eff values (product of protein concentrations and their k on) for nine different protein assembly mechanisms (sequential—Seq; preferentially random—PR; random—Ran; on chromatin—Chr; nucleoplasmic—Nuc) involving 5 proteins as indicated. In all instances K eq = 109 M (here defined as kon/koff; koff is variable depending on the value of k on eff). The rate constant for the final irreversible acetylation of lysine 9 and lysine 14 k mod = 60 min-1, i.e. much faster than the dissociation constant. C. Fraction of promoter binding at equilibrium as function of the k on eff for the same set of mechanisms. In all simulations, the RE became ready for complex assembly at time zero. All models were simulated so as to be at equilibrium prior to time zero. At time zero the RE becomes available for binding. (PDF) [file pcbi.1004236.s004.pdf]

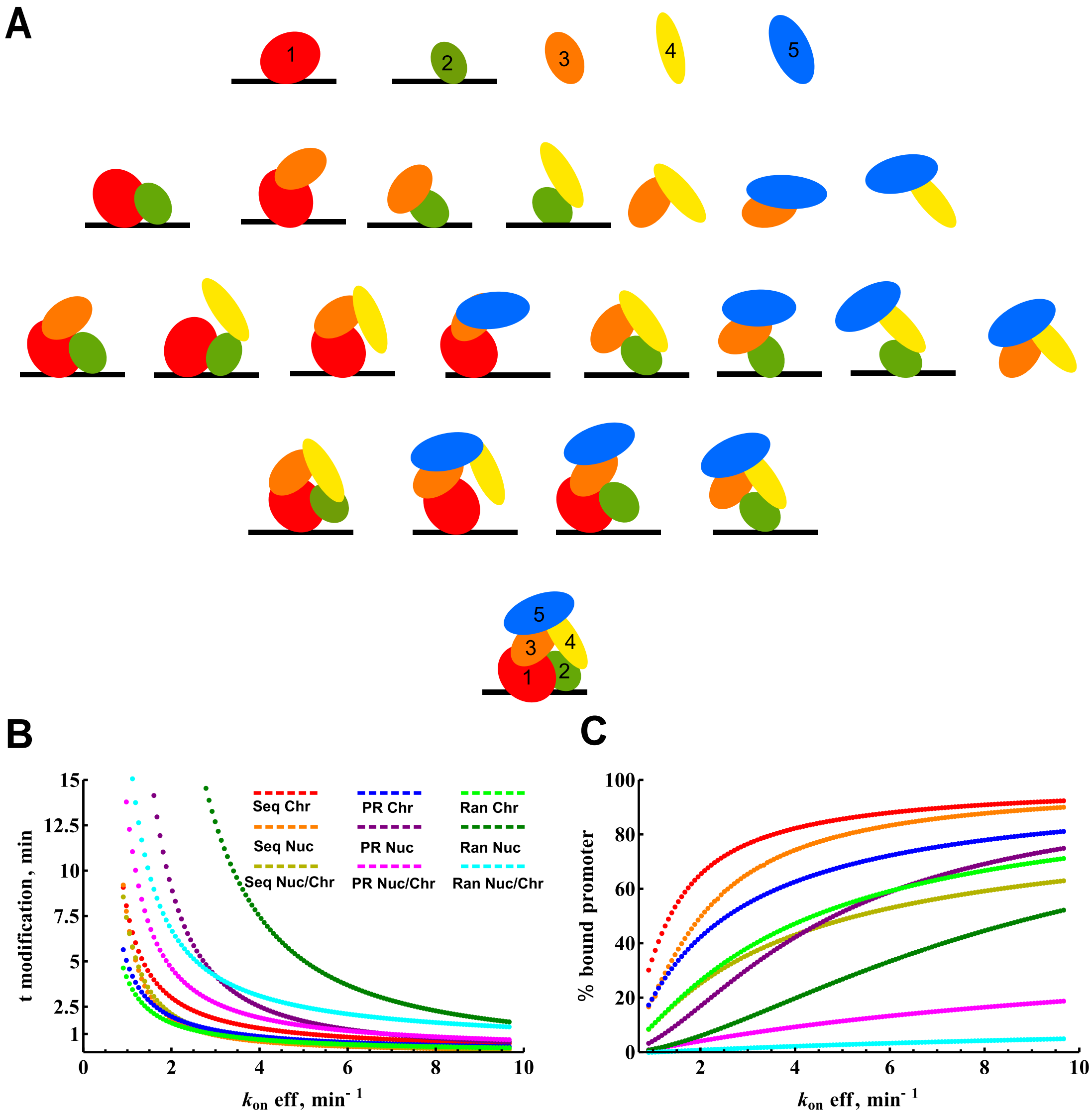

S4 Fig: Mechanisms of protein complex assembly and their duration depending on the sequence and association rate constant. **A.** All complexes that can be formed in the $N = 5$ random mechanism (both in RE and in cytoplasm) that is used in the models are presented; the complexes that can bind RE are shown as bound forms. The lists of complexes that are used in preferentially random and sequential assembly mechanism are given in S2 Table. **B**. Half-time of promoter modification for a range of k_{on}^{eff} values (product of protein concentrations and their k_{on}) for nine different protein assembly mechanisms (sequential – Seq; preferentially random – PR; random – Ran; on chromatin – Chr; nucleoplasmic – Nuc) involving 5 proteins as indicated. In all instances $K_{eq} = 10^9$ M (here defined as k_{on}/k_{off}; k_{off} is variable depending on the value of k_{on}^{eff}). The rate constant for the final irreversible acetylation of lysine 9 and lysine 14 $k_{mod} = 60$ min^{-1}, i.e. much faster than the dissociation constant. **C**. Fraction of promoter binding at equilibrium as function of the k_{on}^{eff} for the same set of mechanisms. In all simulations, the RE became ready for complex assembly at time zero. All models were simulated so as to be at equilibrium prior to time zero. At time zero the RE becomes available for binding.
