# Supplementary material for: Multiplex Eukaryotic Transcription (In)activation: Timing, Bursting and Cycling of a Ratchet Clock Mechanism
Source: PLoS Comput Biol. 2015 Apr 24;11(4):e1004236. doi: 10.1371/journal.pcbi.1004236 (PMC4409292; doi:10.1371/journal.pcbi.1004236)
Supplement: S5 Fig — A. Inactive (unless mass action irreversible) but then irrevertible; B. active, but irrevertible; C. active, forward hesitant (unless mass action irreversible) but then irrevertible, because of hesitation on the reverse route; D. Active, forward hesitation removed, but again irrevertible; E. Active, revertible; but with re-emerging hesitation problem. (PDF) [file pcbi.1004236.s005.pdf]

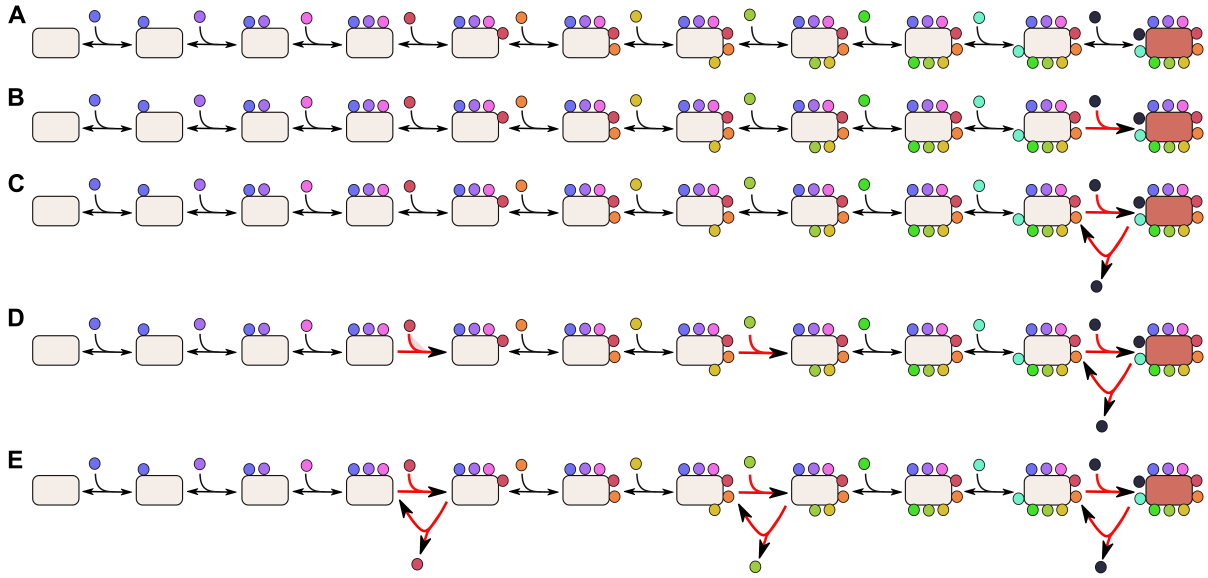

S5 Fig: Various mechanisms for linear transcription mechanism by 10 TFs, with their efficacy. **A**. Inactive (unless mass action irreversible) but then irrevertible; **B**. active, but irrevertible; **C.** active, forward hesitant (unless mass action irreversible) but then irrevertible, because of hesitation on the reverse route; **D.** Active, forward hesitation removed, but again irrevertible; **E**. Active, revertible; but with re-emerging hesitation problem.
