# Supplementary material for: Multiplex Eukaryotic Transcription (In)activation: Timing, Bursting and Cycling of a Ratchet Clock Mechanism
Source: PLoS Comput Biol. 2015 Apr 24;11(4):e1004236. doi: 10.1371/journal.pcbi.1004236 (PMC4409292; doi:10.1371/journal.pcbi.1004236)
Supplement: S6 Fig — Chromatin state transition scheme for the models presented—a basic 9-state promoter model (also used for simulating the Metivier et al. and Karpova et al. data) (A), a modified model with looping between distant RE and TSS (B) used to simulate the Saramäki et al. data. The chromatin states in which RNA polymerase II binding/mRNA production occurs are marked in colour. In scheme A, PR5 is considered in ON state for generic 9-state chromatin model, while for simulating Karpova et al. PR1+PR2 are considered permissive. Specific chromatin states plotted in Figs 4 and 6 in the main text are described in S6 Table. In every model a single chromatin transition corresponds to formation of an N = 5 protein complex on the PR/RE by preferentially random mechanism followed by a chromatin modification step (C) or an N = 4 protein complex on TSS likewise followed by a chromatin modification step (E). For simulating Karpova et al. complexes formed on PR2 contain polymerase and lead to transition to PR3 (D); likewise, PR3 can be bound to the same RNA polymerase II containing complex and its own de-activation complex. Each successful formation of an RNA polymerase II complex leads to the start of elongation. In model B (simulation of Saramäki et al.) the loop is formed between partially bound RE3 and RNA polymerase II bound to TSS3, leading to modification of polymerase that is ready for elongation (F). In all models elongation is modeled as a 30-step process that leads to formation of mature mRNA and release of the RNA polymerase II. (PDF) [file pcbi.1004236.s006.pdf]

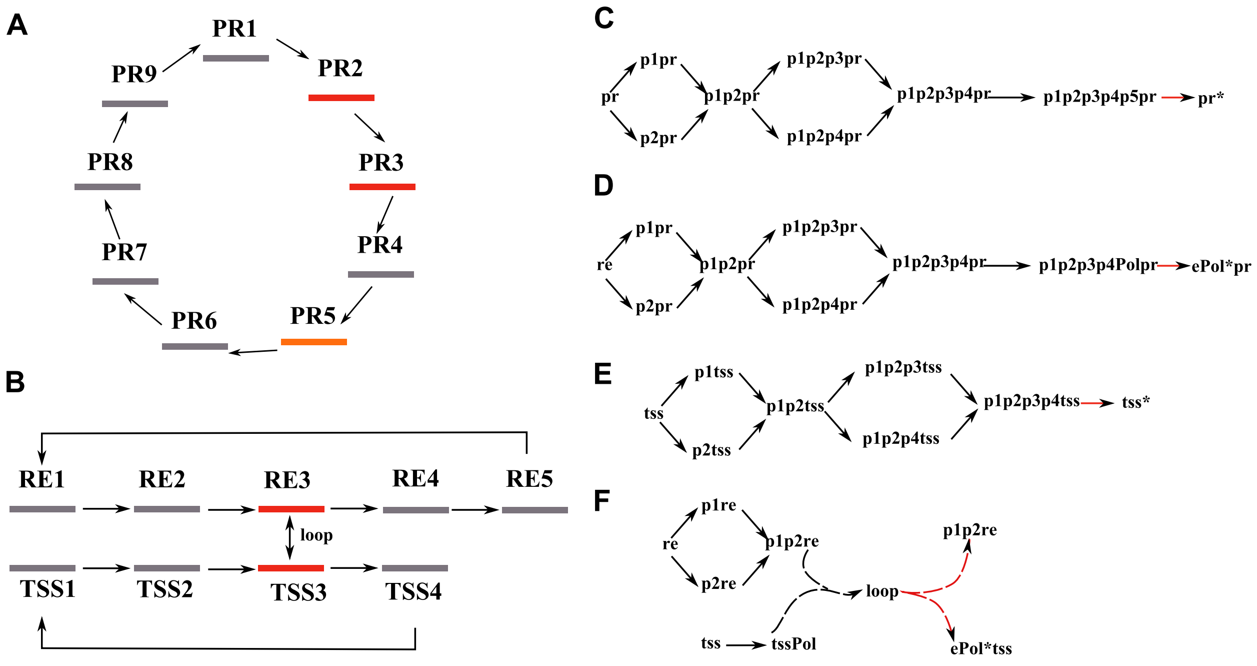

S6 Fig: Structures of detailed initiation models. Chromatin state transition scheme for the models presented – a basic 9-state promoter model (also used for simulating the Metivier *et al.* and Karpova *et al.* data) (**A**), a modified model with looping between distant RE and TSS (**B**) used to simulate the Saramäki *et al.* data. The chromatin states in which RNA polymerase II binding/mRNA production occurs are marked in colour. In scheme **A,** PR5 is considered in ON state for generic 9-state chromatin model, while for simulating Karpova *et al.* PR1+PR2 are considered permissive. Specific chromatin states plotted in Figs. 4 and 6 in the main text are described in S6 Table. In every model a single chromatin transition corresponds to formation of an N = 5 protein complex on the PR/RE by preferentially random mechanism followed by a chromatin modification step (**C**) or an N = 4 protein complex on TSS likewise followed by a chromatin modification step (**E**). For simulating Karpova *et al.* complexes formed on PR2 contain polymerase and lead to transition to PR3 (**D**); likewise, PR3 can be bound to the same RNA polymerase II containing complex and its own de-activation complex. Each successful formation of an RNA polymerase II complex leads to the start of elongation. In model **B** (simulation of Saramäki *et al.*) the loop is formed between partially bound RE3 and RNA polymerase II bound to TSS3, leading to modification of polymerase that is ready for elongation (**F**). In all models elongation is modeled as a 30-step process that leads to formation of mature mRNA and release of the RNA polymerase II.
