# Supplementary material for: Multiplex Eukaryotic Transcription (In)activation: Timing, Bursting and Cycling of a Ratchet Clock Mechanism
Source: PLoS Comput Biol. 2015 Apr 24;11(4):e1004236. doi: 10.1371/journal.pcbi.1004236 (PMC4409292; doi:10.1371/journal.pcbi.1004236)
Supplement: S1 Table — A realistic interaction scheme for a complex of five proteins is presented in the form of an interaction matrix. The interaction matrix was used to produce the mass balance and rate vectors for the random assembly model. (PDF) [file pcbi.1004236.s007.pdf]

| | re | p1 | p2 | p3 | p4 | p5 |
|---|---|---|---|---|---|---|
| **re** | 0 | **1** | **1** | 0 | 0 | 0 |
| **p1** | **1** | 0 | **1** | **1** | 0 | 0 |
| **p2** | **1** | **1** | 0 | **1** | **1** | 0 |
| **p3** | 0 | **1** | **1** | 0 | **1** | **1** |
| **p4** | 0 | 0 | **1** | **1** | 0 | **1** |
| **p5** | 0 | 0 | 0 | **1** | **1** | 0 |

**S1 Table: Interaction matrix of protein complexes.** A realistic interaction scheme for a complex of five proteins is presented in the form of an interaction matrix. The interaction matrix was used to produce the mass balance and rate vectors for the random assembly model.
