# Supplementary material for: Multiplex Eukaryotic Transcription (In)activation: Timing, Bursting and Cycling of a Ratchet Clock Mechanism
Source: PLoS Comput Biol. 2015 Apr 24;11(4):e1004236. doi: 10.1371/journal.pcbi.1004236 (PMC4409292; doi:10.1371/journal.pcbi.1004236)
Supplement: S2 Table — A customary algorithm was used to remove reactions involving complexes formed only in fully random mechanisms. (PDF) [file pcbi.1004236.s008.pdf]

	N=2	N=3	N=4	N=5
**Preferentially random RE-bound**	p1p2r1	p1p2p3r1<br>p1p2p4r1	p1p2p3p4r1<br>p1p2p3p5r<br>p1p2p4p5r1	p1p2p3p4p5r1
**Preferentially random nuclear**	p1p2<br>p3p4<br>p3p5<br>p4p5	p1p2p3<br>p1p2p4	p1p2p3p4<br>p1p2p3p5<br>p1p2p4p5	p1p2p3p4p5
**Sequential RE-bound**	p1p2r1	p1p2p3r1	p1p2p3p4r1	p1p2p3p4p5r1
**Sequential nuclear**	p1p2	p1p2p3	p1p2p3p4	p1p2p3p4p5

S2 Table: Complexes formed in preferential random and sequential assembly mechanisms. A customary algorithm was used to remove reactions involving complexes formed only in fully random mechanisms.
