# Supplementary material for: Multiplex Eukaryotic Transcription (In)activation: Timing, Bursting and Cycling of a Ratchet Clock Mechanism
Source: PLoS Comput Biol. 2015 Apr 24;11(4):e1004236. doi: 10.1371/journal.pcbi.1004236 (PMC4409292; doi:10.1371/journal.pcbi.1004236)
Supplement: S3 Table — A customary algorithm was used to calculate rate constants for the corresponding protein association and dissociation reactions. (PDF) [file pcbi.1004236.s009.pdf]

Name	MW,<br>kDa	R,<br>μm	D,<br>$\mu m^2/s$	$k_{on}/100$,<br>s^{-1}	$k_{on}/100$,<br>$pM^{-1}\ s^{-1}$	Kd,<br>pM	$k_{off,}$<br>s^{-1}	[C],<br>pM
re	**-**	**0.0006**	**0**	*re-p1*<br>**$1.1*10^{-5}$**	*re-p1*<br>**$0.7*10^{-5}$**	*re-p1*<br>**10^3**	*re-p1*<br>**$0.7*10^{-2}$**	**1.4**
p1	**50**	**0.025**	**23.2**	*p1-p2p3*<br>**$3.7*10^{-5}$**	*p1-p2p3*<br>**$2.2*10^{-5}$**	*p1-p2p3*<br>**10^2**	*p1-p2p3*<br>**$2.2*10^{-3}$**	**140-<br>14,000**
p2p3	**100**	**0.031**	**29.3**	*p2p3-re*<br>**$1.1*10^{-5}$**	*p2p3-re*<br>**$0.7*10^{-5}$**	*p2p3-re*<br>**10^3**	*p2p3-re*<br>**$0.7*10^{-2}$**	**-**

S3 Table: Example of parameter calculation. A customary algorithm was used to calculate rate constants for the corresponding protein association and dissociation reactions.
