# Supplementary material for: Multiplex Eukaryotic Transcription (In)activation: Timing, Bursting and Cycling of a Ratchet Clock Mechanism
Source: PLoS Comput Biol. 2015 Apr 24;11(4):e1004236. doi: 10.1371/journal.pcbi.1004236 (PMC4409292; doi:10.1371/journal.pcbi.1004236)
Supplement: S4 Table — Protein association and dissociation rate constants and concentrations were calculated in the same way as shown in S3 Table. The modification rate constants were chosen in a way to provide the same effective rates as a single protein-binding step to reduce the noise in waiting times. The values are in the range for kcat of chromatin modifying enzymes measured in vitro [s35-37]. Elongation and export times were estimated from data available in literature [s38-41] and modeled as a multi-step process (N = 30). A 30 min life-time was assumed; degradation was modeled as multi-step process with (N = 20). For models used to simulate data, additional parameters as well as parameters different from the main model are given. For simulation of the Karpova et al. data, the initiation rate constant was taken to be faster than the RNA polymerase II off-rate. The rates of mRNA elongation (modeled as N = 30 process) and degradation (modeled as N = 20 process) were adjusted to fit the data. (PDF) [file pcbi.1004236.s010.pdf]

Constant	Value	Units
k_{on} p1/p2 step1	$6*10^{-4}$	min^{-1}
k_{off} p1/p2 step1	$4.5*10^{-1}$	min^{-1}
k_{on} p1/p2 step2	$1*10^{-3}$	min^{-1}
k_{off} p1/p2 step2	$7.3*10^{-1}$	min^{-1}
k_{on} p3/p4 1 step1	$1.14*10^{-3}$	min^{-1}
k_{off} p3/p4 1 step1	$8.4*10^{-1}$	min^{-1}
k_{on} p3/p4 2 step2	$1.2*10^{-3}$	min^{-1-}
k_{off} p3/p4 2 step2	$9.2*10^{-1}$	min^{-1}
k_{on} p5	$1.32*10^{-3}$	min^{-1}
k_{off} p5	$9.8*10^{-1}$	min^{-1}
k_{mod}	1.36	min^{-1}
k_{ini}	60	min^{-1}
1 step k $mRNA_{prod}$	2	min^{-1}
1 step k $mRNA_{deg}$	$6.6*10^{-1}$	min^{-1}
protein total	$1.5*10^{3}$	number of molecules
pol	$2*10^{4}$	number of molecules
promoter	1	number of molecules
**Parameters adjusted for the Metivier *et al.* data simulation**		
protein total	$1.8*10^{3}$	number of molecules
**Parameters for adjusted the Karpova *et al.* data simulation**		
1 step k $mRNA_{prod}$	$3*10^{1}$	min^{-1}
1 step k $mRNA_{deg}$	$2*10^{-1}$	min^{-1}
total protein binding to PR3	$3*10^{3}$	number of molecules
total protein binding to PR2, including pol	$1.5*10^{4}$	number of molecules

S4 Table: Parameters for transcription in 9-state promoter model. Protein association and dissociation rate constants and concentrations were calculated in the same way as shown in S3 Table. The modification rate constants were chosen in a way to provide the same effective rates as a single protein-binding step to reduce the noise in waiting times. The values are in the range for k_{cat} of chromatin modifying enzymes measured *in vitro* [s35-37]. Elongation and export times were estimated from data available in literature [s38-41] and modeled as a multi-step process (N=30). A 30 min life-time was assumed; degradation was modeled as multi-step process with (N=20). For models used to simulate data, additional parameters as well as parameters different from the main model are given. For simulation of the Karpova *et al.* data, the initiation rate constant was taken to be faster than the RNA polymerase II off-rate. The rates of mRNA elongation (modeled as N=30 process) and degradation (modeled as N=20 process) were adjusted to fit the data.
