# Supplementary material for: Multiplex Eukaryotic Transcription (In)activation: Timing, Bursting and Cycling of a Ratchet Clock Mechanism
Source: PLoS Comput Biol. 2015 Apr 24;11(4):e1004236. doi: 10.1371/journal.pcbi.1004236 (PMC4409292; doi:10.1371/journal.pcbi.1004236)
Supplement: S5 Table — Protein association and dissociation rate constants and concentrations were calculated in the same way as shown in S3 Table. Only the new mRNA synthesis was modelled. As the number of states is lower in this model, a lower degree of reversibility is required to reach comparable passive synchronization as in the 9-state model, hence the lower off rates. The on-rate of loop formation was taken as an estimation from data presented by Polikanov et al. (2007) [s42], which suggests that the average rate for DNA loop formation of comparable length in vitro is at least faster than 60 seconds. The loop was assumed to be stable (Kd = 1012) due to a big protein interaction surface. The polymerase modification step and promoter step were modelled explicitly. The protein concentrations were adjusted to fit experimental data. (PDF) [file pcbi.1004236.s011.pdf]

Constant	Value	Units
k_{on} p1/p2 step1	$6*10^{-4}$	min^{-1}
k_{off} p1/p2 step1	$6*10^{-2}$	min^{-1}
k_{on} p1/p2 step2	$1*10^{-3}$	min^{-1}
k_{off} p1/p2 step2	10^{-1}	min^{-1}
k_{on} p3/p4 1 step1	$1.14*10^{-3}$	min^{-1}
k_{off} p3/p4 1 step1	$1.14*10^{-1}$	min^{-1}
k_{on} p3/p4 2 step2	$1.2*10^{-3}$	min^{-1-}
k_{off} p3/p4 2 step2	$1.2*10^{-1}$	min^{-1}
k_{on} p5	$1.32*10^{-3}$	min^{-1}
k_{off} p5	$1.32*10^{-1}$	min^{-1}
k_{mod}	$7.8*10^{-1}$	min^{-1}
k $loop_{on}$	$7.8*10^{-1}$	min^{-1}
k $loop_{off}$	$7.8*10^{-2}$	min^{-1}
k_{on} pol	$6*10^{-2}$	min^{-1}
k_{off} pol	$6*10^{-1}$	min^{-1}
$k_{pol\ mod}$	1.6	min^{-1}
k_{esc}	$6*10^{-1}$	min^{-1}
protein total	450	number of molecules
re/tss	1	number of molecules
pol	10^4	number of molecules

S5 Table: Parameters for the promoter model with looping between RE and TSS for simulating data from Saramäki *et al.* Protein association and dissociation rate constants and concentrations were calculated in the same way as shown in S3 Table. Only the new mRNA synthesis was modelled. As the number of states is lower in this model, a lower degree of reversibility is required to reach comparable passive synchronization as in the 9-state model, hence the lower off rates. The on-rate of loop formation was taken as an estimation from data presented by Polikanov et al. (2007) [s42], which

suggests that the average rate for DNA loop formation of comparable length *in vitro* is at least faster than 60 seconds. The loop was assumed to be stable ($K_d = 10^{12}$) due to a big protein interaction surface. The polymerase modification step and promoter step were modelled explicitly. The protein concentrations were adjusted to fit experimental data.
