# Supplementary material for: Multiplex Eukaryotic Transcription (In)activation: Timing, Bursting and Cycling of a Ratchet Clock Mechanism
Source: PLoS Comput Biol. 2015 Apr 24;11(4):e1004236. doi: 10.1371/journal.pcbi.1004236 (PMC4409292; doi:10.1371/journal.pcbi.1004236)
Supplement: S6 Table — Specifications (structures and parameters) of each model are described, and the model states plotted in the main text figures are listed. (PDF) [file pcbi.1004236.s012.pdf]

| **Model (main text figure)** | **Model scheme** | **model parameters** | **Plotted promoter/mRNA states** |
|---|---|---|---|
| 9-state promoter model (Figs. 4 and 5) | S6A and S6C Figs | S4 Table | State pr2 (blue), pr4 (green), pr6 (red), pr8 (orange) (Figs. 4C and D);<br>Sum of mRNA [31-49] (Fig. 5C-F) |
| Simulation of Metivier *et al.* data (Fig. 6A) | S6A and S6C Figs | S4 Table | Sum p4 bound pr2 (purple), p3 bound pr3 (dark blue), sum all pr7 states (green), sum all pr7 and pr8 states (green), sum all pr5 states (red), sum all pr4, pr5 and pr6 states (blue) |
| Simulation of Karpova *et al.* data (Fig. 6C) | S6A and S6D Figs | S4 Table | mRNA[2] |
| Simulation of Saramäki *et al.* data (Fig. 6E) | S6B, S6E and S6F Figs | S5 Table | Loop (black), tss3-epol (red) |

**S6 Table: Summary of information on different promoter models.** Specifications (structures and parameters) of each model are described, and the model states plotted in the main text figures are listed.
