# Supplementary material for: Multiplex Eukaryotic Transcription (In)activation: Timing, Bursting and Cycling of a Ratchet Clock Mechanism
Source: PLoS Comput Biol. 2015 Apr 24;11(4):e1004236. doi: 10.1371/journal.pcbi.1004236 (PMC4409292; doi:10.1371/journal.pcbi.1004236)
Supplement: S1 Text — (PDF) [file pcbi.1004236.s013.pdf]

S1 Text: Promoter sensitivity to multiple TFs in the equilibrium binding model with and without cooperativity

We demonstrate first that a simple design for gene regulation, based on independent equilibrium binding of regulatory factors to DNA/chromatin sites cannot function properly for eukaryotic genes. According to such an equilibrium-binding mechanism, individual binding sites for TFs and their co-regulators saturate individual binding sites in a concentration-dependent manner. For a single TF, the transcription rate v depends on its concentration, T, as:

$$v = k \cdot f(\boldsymbol{x}) \cdot \frac{T}{T+K_D} = k \cdot f(\boldsymbol{x}) \cdot \frac{1}{1+K_D'} = k' \cdot \phi(T) \quad (1)$$

Here "k" denotes the transcription rate constant, "K_D" the affinity constant of the chromatin site for the TF and "$f(\boldsymbol{x})$" a function that describes the effect of other factors, such as RNA polymerase and other proteins. We define the apparent transcription rate constant k' as $k \cdot f(\boldsymbol{x})$. The factor $\phi(T)$ denotes the saturation degree of the site with the TF. K_D' is the effective concentration-independent dissociation constant, which equals the first order dissociation rate constant (k_B) divided by the quasi first-order association rate constant (k_F'), $K_D' = \frac{K_D}{T} = \frac{k_B}{k_F T} = \frac{k_B}{k_F'}$.

For n TFs, the saturation of the gene $\Phi(\boldsymbol{T})$ is defined as the product of the saturation of the individual TF binding sites,

$$\Phi(\boldsymbol{T}) \equiv \frac{v}{k'} = \prod_{i=1}^{n} \frac{1}{1+K_{D,i}'} = \prod_{i=1}^{n} \phi_i(T_i) \quad (2)$$

This equation illustrates that saturation of each site by 50% leads to a much lower saturation degree for the gene:

$$\Phi(\boldsymbol{T}) < \left(\frac{1}{2}\right)^n \ll \frac{1}{2}.$$

In order to achieve 75% of maximal gene activity, i.e. $\Phi(\boldsymbol{T}) = 0.75$, the binding process would need to be effectively irreversible: in case the individual binding sites are equally saturated and $n = 12$, the saturation degree of a single site should equal 98%, i.e. $\phi_i(T_i) = \sqrt[12]{0.75} = 0.98$. This means that K_D' should equal ~0.02. Thus, the TF concentration should exceed the K_D by a factor of 50, which is unrealistic. The tendency of requiring enhanced saturation of individual binding sites is shown in Fig. 1A in the main text.

There exists also another limitation of equilibrium-binding models of gene regulation with many regulatory factors. The controllability of gene activity by TFs quickly reduces when many factors are involved. The sensitivity of the transcription rate to a specific factor depends on the extent of saturation of the gene with that factor,

$$\frac{\partial \ln v}{\partial \ln T_i} = 1 - \phi_i(T_i) \tag{3}$$

Equation 3 indicates that the saturation degree of individual sites, ϕ_i, increases with the number of regulatory factors to achieve the same net degree of saturation of the entire gene, Φ. Relationship 3 shows that, in addition, the gene then loses sensitivity to regulators. Thus, genes become progressively harder to regulate when the number of regulatory factors increases.

One would suspect that cooperativity among the TFs would reduce the effects on the reduction of the promoter sensitivity to individual TFs with their increasing numbers. We will demonstrate that in principle it can do so, but the interaction coefficients between TFs would have to increase considerably with the number of regulatory factors, which would again lead to an unrealistic scenario. This we will show with a simple (extreme) model for cooperative regulation by regulatory factors. Consider for instance the simplest cooperative binding kinetics among *n* regulatory factors,

$$\Phi(\mathbf{T}, n, \beta) = \frac{\frac{\alpha^n}{\beta^{n-1}}}{1+\sum_{i=1}^{n}\binom{n}{i}\frac{\alpha^i}{\beta^{i-1}}} \tag{4}$$

where $\alpha = T_i / K_i$ (for all i) and β is an interaction coefficient between regulatory factors that changes the affinity constant of one regulator when another regulator is bound to its DNA site by factor β. Positive cooperativity occurs when $\beta < 1$. For instance, for two factors we obtain,

$$\Phi(\mathbf{T}, 2, \beta) = \frac{\frac{T_1 T_2}{\beta K_1 K_2}}{1 + \frac{T_1}{K_1} + \frac{T_2}{K_2} + \frac{T_1 T_2}{\beta K_1 K_2}} \tag{5}$$

Cooperativity is absent when $\beta = 1$ and the previous model (equation 5) is obtained. The interaction coefficient β can be equated in terms of a Gibbs free energy for interaction between regulators. To assess the performance of cooperativity we determined what the value of the interaction coefficient β should be, such that the degree of saturation of the entire regulatory system $\Phi(\mathbf{T}, n, \beta)$ equals that of its single sites, $\phi(T)$. We achieved this by calculating the optimal value of β such that the squared difference between $\Phi(\mathbf{T}, n, \beta)$ and $\phi(T)$ is minimal at a given number of TFs, *n*, for a range of values of α (from 0 to 20, in steps $\Delta\alpha$ of 0.5). S1 Fig. indicates that the optimal value for the interaction coefficient β drops sharply with *n*, indicating that increasing levels of cooperativity is required to compensate for the loss in saturation degree in $\Phi(\mathbf{T}, n, \beta)$ at higher numbers of TFs. The decrease in equilibrium binding constant required in case of smaller number of TFs is in the order of 2 to 8 times, which is compatible with the values found experimentally [s1-3]. For a number of TFs of 8

or higher, however, the levels of decrease in binding constant become unrealistically high (10 time or higher). Even though we cannot rule out that such cooperative mechanisms can exist we deem it unlikely. Moreover, the scenario we sketched gives a very optimistic estimate, as this model does assume a severe cooperativity: all TFs cooperatively interact with each other. In the case that fewer TFs interact, even lower values of β are anticipated. This would indicate even higher free energies for stabilization of the DNA bound states.
