# Supplementary material for: Multiplex Eukaryotic Transcription (In)activation: Timing, Bursting and Cycling of a Ratchet Clock Mechanism
Source: PLoS Comput Biol. 2015 Apr 24;11(4):e1004236. doi: 10.1371/journal.pcbi.1004236 (PMC4409292; doi:10.1371/journal.pcbi.1004236)
Supplement: S2 Text — (PDF) [file pcbi.1004236.s014.pdf]

S2 Text: Mean time of protein complex assembly using reversible versus irreversible mechanisms

For a sequential non-branched protein assembly mechanism (S3 Fig.) the mean first passage time equals the sum of the mean time to complete individual steps. Hence in case when all rate constants are equal:

$$\tau_{irr}(n) = \frac{n}{k_f} \tag{1}$$

Where n is the number of proteins and k_f the association constant. Deriving the mean first passage time when the proteins can dissociate is less trivial. To this end we first employed the method described by Dobrzyński and Bruggeman (2009) [s4]. It allows calculating moments of the assembly time distributions for an arbitrary mechanism by using the Laplace-transformed ordinary differential equation (ODE) system. We obtained assembly means for a strict sequential mechanism with 3 to 5 proteins:

$$\tau_{rev}(3) = \frac{3k_f^2 + 2k_f k_b + k_b^2}{k_f^3} = \frac{3}{k_f} + \frac{1}{k_f}\left(2\frac{k_b}{k_f} + \frac{k_b^2}{k_f^2}\right) \tag{2}$$

$$\tau_{rev}(4) = \frac{4k_f^3 + 2k_f k_b^2 + 3k_b k_f^2 + k_b^3}{k_f^4} = \frac{4}{k_f} + \frac{1}{k_f}\left(3\frac{k_b}{k_f} + 2\frac{k_b^2}{k_f^2} + \frac{k_b^3}{k_f^3}\right) \tag{3}$$

$$\tau_{rev}(5) = \frac{5k_f^4 + 4k_b k_f^3 + 3k_b^2 k_f^2 + 2k_f k_b^3 + k_b^4}{k_f^5} = \frac{5}{k_f} + \frac{1}{k_f}\left(4\frac{k_b}{k_f} + 3\frac{k_b^2}{k_f^2} + 2\frac{k_b^3}{k_f^3} + \frac{k_b^4}{k_f^4}\right) \tag{4}$$

Thus, by induction, the general expression for sequential reversible assembly of n proteins given equal forward and reverse rate constants is:

$$\tau_{rev}(n) = \frac{n}{k_f} + \sum_{i=1}^{n-1} \frac{i}{k_f}\left(\frac{k_b}{k_f}\right)^{n-i} = \frac{n}{k_f} + \sum_{i=1}^{n-1} \frac{i}{k_f}(K_D)^{n-i} \tag{5}$$

The ratio of assembly times between the reversible and irreversible mechanism is given by:

$$\frac{\tau_{rev}(n)}{\tau_{irr}(n)} = 1 + \sum_{i=1}^{n-1} \frac{i}{n}(K_D)^{n-i} \tag{6}$$

To evaluate the ratio for e.g. branched mechanisms we applied the same Laplace transform method to ODE systems describing the n = 3 complex formation for one fully random and two partially random assembly schemes – with branching in the beginning and at the end (S3 Fig). The mean assembly times are as follows:

$$\tau_{rev}(3, branch\ start) = \frac{5k_f^2 + 5k_f k_b + 2k_b^2}{2k_f^3} = \frac{5}{2k_f} + \frac{1}{k_f}\left(\frac{5}{2}K_D + K_D^2\right) \tag{7}$$

$$\tau_{rev}(3, branch\ end) = \frac{5k_f^2 + 2k_f k_b + k_b^2}{2k_f^3} = \frac{5}{2k_f} + \frac{1}{k_f}\left(K_D + \frac{1}{2}K_D^2\right) \tag{8}$$

$$\tau_{irr}(3, branch\ start) = \tau_{irr}(3, branch\ end) = \frac{5}{2k_f} \qquad (9)$$

$$\tau_{rev}(3, random) = \frac{11k_f^2 + 7k_f k_b + 2k_b^2}{6k_f^3} == \frac{11}{6k_f} + \frac{1}{k_f}\left(\frac{7}{6}K_D + \frac{1}{3}K_D^2\right) \qquad (10)$$

$$\tau_{irr}(3, random) = \frac{11}{6k_f} \qquad (11)$$

The ratios were calculated for three branched and one fully sequential mechanism and plotted against K_D (S3 Fig). For the inset, the probability density distribution of the random n = 3 assembly mechanism found numerically for $k_f = 10$ and $k_b = 1$ by the same Laplace method was plotted.
