# Supplementary material for: Multiplex Eukaryotic Transcription (In)activation: Timing, Bursting and Cycling of a Ratchet Clock Mechanism
Source: PLoS Comput Biol. 2015 Apr 24;11(4):e1004236. doi: 10.1371/journal.pcbi.1004236 (PMC4409292; doi:10.1371/journal.pcbi.1004236)
Supplement: S3 Text — (PDF) [file pcbi.1004236.s015.pdf]

S3 Text: Timing of the protein assembly mechanisms

As little is known about the mechanism of assembly of the protein sub-complexes involved in transcription, we chose to compare the kinetics of various alternative mechanisms of complex assembly to identify mechanisms that operate in experimentally determined time-scale and protein-concentration regimes. We distinguished three mechanisms with respect to the order of assembly: i) random (Ran), with no specific binding order, ii) preferentially random (PR) with partially determined order, and iii) sequential (Seq) with a uniquely defined order. For each of these mechanisms we also considered three assembly locations: i) exclusively on the chromatin (Chr), ii) only in the nucleoplasm with the final complex binding to chromatin (Nuc), or iii) both in the nucleoplasm and on the chromatin (Nuc/Chr). Random-mechanism models were generated using an interaction matrix (S1 Table). A realistic scenario was considered given physical constraints of binding surfaces: firstly, two proteins interact directly with the RE; each protein has 2 to 4 (on average 3) binding partners. A biological example of such a complex could be the DNA-interacting PPARβ/δ-RXR heterodimer bound to scaffolding co-factor PGC1α and chromatin-modifying enzymes CBP and SRC1 [49]. An extra algorithm was applied to remove the reactions involving RE-bound or free complexes to obtain the random one in the nucleoplasm and on the RE, respectively.

For the random mechanism all possible complexes allowed by the interaction matrix are formed, resulting in a total of 40 complexes. To produce the mass balance and rate vectors for the preferential random and the sequential mechanisms a customary algorithm was used to remove reactions involving complexes formed only in the fully random mechanism (S2 Table). For preferential random/sequential RE and preferential random/sequential nuclear interactions only complexes on the RE and in the nucleus, respectively, were allowed.

We have tested all nine possible models for realistic parameter ranges. Detailed information on the protein concentrations and binding rate constants calculation can be found in S3 Table. To determine the on-rate constants, first the diffusion limited ones were calculated by using the Einstein-Smoluchowski relation [6, s5]. The monomer proteins were considered to have a molecular weight (MW) of 50 kD; two-times higher or lower values do not have a significant effect on the outcome. The Stokes radii (R) were calculated using a standard curve (radius versus MW) measured by Ribbeck and Gorlich (2001) [s6]. The diffusion coefficients (D) were calculated by the Smoluchowski formula using as standard measured green fluorescent protein diffusion coefficient in the nucleoplasm [s5, s7, s8]. The real on-rate constants are, however, likely to be lower. There are no direct measurements of association rate constants (k_{on}) available, thus we used the equilibrium constants and off rates for estimation purposes.

For ODE simulation the parameters were used in pM units; for stochastic simulation in molecules/pL units. The off-rates were calculated accordingly depending on the equilibrium constant.

The measurements available for the dissociation rate constants (k_{off}) of proteins from chromatin, both *in vivo* and *in vitro* have been shown to be of the order of 10^{-2} s^{-1} [42, 43, s9], corresponding to dwell times of around a minute. It is known that relatively strong non-covalent protein interactions lie in the range of 10^{8} to 10^{10} M [s10]. As a reasonable approximation we took the average K_D = 10^{9} M, corresponding well with some of the available *in vitro* measurements [s11].

Given the 10^{9} M equilibrium constant and 10^{-2} s^{-1} off rate constant, the on rate constant should be in the range of 10^{7} $M^{-1}s^{-1}$ corresponding to being ~100-times lower than the diffusion limit of 10^{9} $M^{-1}s^{-1}$. This corresponds well with previous findings [s12-14], as well as the available *in vitro* measurements [42]. Thus we assumed the on rate constants k_{on} in the model to be 100-times lower than the calculated diffusion limited constant. For the majority of reported TF, as well as proteins with histone modifying activity, copy numbers are in the range from 10^{3} -10^{4} molecules per cell (see S2 Fig., based on the data from [38-41]). The cell nucleus can be estimated to be 1.2 pL given the measurement of cell volume (3-4 pL [36]) and nucleo-cytoplasmic ratio (1:3 [37]) giving a concentration of 10^{-9} to 10^{-8} M. Considering the possibility that actual concentrations are lower due to a fraction the TF molecules being bound, we considered a range of 10^{2} to the top estimates of 10^{4}, yielding an effective k_{on} range between 0.1 min^{-1} and 10 min^{-1}. For the fixing irreversible step, we considered the apparent first-order chromatin modification reaction, As an estimate of its rate constant k_{mod} we took the reported k_{cat} 1 s^{-1} of chromatin modifiers [s15, s16]. This makes the whole process hit-and-run with the overall rate being determined by the above mean *first* assembly time calculations.

We calculated the (average) half-time for protein complex formation followed by a single histone modification capture event for these different mechanisms. We used a range of realistic effective on-rate constant values, while adjusting the k_{off} values such that the equilibrium constant remained 10^{9} M (S4B and S4C Fig.). We calculated the completion time. In the case of assembly on chromatin, the time of formation increased with assembly randomness, while for mechanisms involving assembly in the nucleoplasm the trend was opposite. For sequential mechanisms, nucleoplasmic assembly was faster than both assembly on chromatin and mixed assembly, while for the (preferentially) random mechanism assembly in the nucleoplasm was the slowest. These results are explained by two effects with opposite trends. First, random assembly schemes tend to be faster as they have more assembly paths, whereas ordered sequential schemes will have many unproductive attempts because components arrive in the wrong order. Secondly, random assembly schemes have many more intermediate complexes that reduce the probability to be in a specific state. In the random mechanism any out of 4 tetramer complexes can precede the ultimate pentamer, but only one in the sequential process. Hence, entropy will tend to keep the random assembly system in the tetrameric state. In case

of assembly on chromatin, the individual states are more probable and in the nucleoplasm-assembly mechanisms free protein concentrations are significantly reduced as a result of complex formation causing slower assembly. For the range of realistic effective on-rate constants, the models give rise to completion times that differ by several orders of magnitude (S4B Fig).

In order to evaluate the performance of the protein complex assembly mechanisms, we also have to consider to what extent they can give rise to promoter saturation (S4C Fig). Preferentially random mixed-nuclear chromatin assembly gives rise to low promoter saturation (<20%) across the entire effective k_{on} range; too much protein is wasted in complexes in the nucleoplasm. S4C and S4B Fig. then indicate that on-chromatin assembly mechanisms are faster and readily saturate the chromatin sites. This is because assembly in the nucleoplasm engages protein complexes super-stoichiometric to the DNA-binding site concentration and hence deplete protein monomers. For this assembly on chromatin, random order is faster but less complete.

The calculations also suggest a trade-off: random order is fast but may not lead to complete saturation of the promoter; ordered-sequential may be 5-times slower but leads to almost complete promoter occupancy.
