# Supplementary material for: Multiplex Eukaryotic Transcription (In)activation: Timing, Bursting and Cycling of a Ratchet Clock Mechanism
Source: PLoS Comput Biol. 2015 Apr 24;11(4):e1004236. doi: 10.1371/journal.pcbi.1004236 (PMC4409292; doi:10.1371/journal.pcbi.1004236)
Supplement: S4 Text — (PDF) [file pcbi.1004236.s016.pdf]

S4 Text: Revertible linear mechanism of transcriptional (in)activation

Genes can be turned OFF in at least two ways: either by following the same mechanism in reverse back to the OFF state or by proceeding along a new path according to the batch-wise mechanism to arrive at the OFF state. The former we will refer to as a (revertible) linear transcriptional mechanism and the latter as the transcriptional cycle mechanism. Here we will discuss in detail different versions of the linear transcriptional mechanism and show that these are unsatisfactory.

One way to make the process of transcriptional activation revertible is to make it thermodynamically reversible. Then all the binding reactions of the individual regulatory factors should be reversible. This corresponds to the equilibrium transcriptional activation mechanism discussed in the main text. S5A Fig. shows this mechanism for the case where ten TFs bind reversibly to the chromatin, producing the activated transcription complex (in red). More than half-reversible transcriptional activation by each of 10 TFs (K_D'<1, see above) would lead to a transcriptional activity of less than 0.5^{10} = 0.1% of maximal. As we showed above, for the transcription complex to be more than 75% active at steady state, the dimensionless dissociation equilibrium constant K_D' should equal 0.028, which means that the transcriptional activation process would be 97% irreversible (through mass action). To achieve this degree of irreversibility at the realistic concentration of TFs and estimated value for the diffusion-limited association rate-constant, the dissociation rate constants k_B would have to equal 2*0.024 = 0.056 min^{-1}. This would indicate that proteins should reside on chromatin for 18 min, much longer than observed experimentally [29]. Since the assembly process is reversible with a bias towards the direction of association, dissociation would then take too long. Thus, the mechanism of S5A Fig. is unfit for revertible transcriptional regulation. S5B Fig. makes the final step irreversible by coupling an exergonic reaction to it. This would increase steady state activation of the transcription complex at more reversible values of the dissociation constant. In this mechanism it would still not be possible to switch gene expression off though, unless a reverse irreversible step was added to the final association reaction (see S5C Fig.). The long chain of reversible reactions preceding the final irreversible activation step would still make transcriptional activation slow (again because of the 'hesitation' phenomenon, see text) unless the reverse rate constants were to be much smaller than the forward ones; yet then it would take a very long time to switch off gene expression. Above we showed that the hesitation problem could be alleviated by operating batch-wise, as illustrated in S5D Fig.: every third reaction is irreversible here, by making its k_B very slow. Because in this mechanism gene expression could not be switched off, we added an antiparallel reverse irreversible process to each irreversible step (S5E Fig.). At each point after an irreversible activation step in this mechanism there would be both a forward and a backward process at high rate: however, this would again lead to hesitation and cause a major delay in the onset of transcriptional activity. We conclude that none of the schemes in S5 Fig. would lead to a strong transcriptional activation rapidly that could

also be switched off rapidly (where rapid means within tens of minutes, as has been found experimentally [27, s17]).
