# Supplementary material for: Multiplex Eukaryotic Transcription (In)activation: Timing, Bursting and Cycling of a Ratchet Clock Mechanism
Source: PLoS Comput Biol. 2015 Apr 24;11(4):e1004236. doi: 10.1371/journal.pcbi.1004236 (PMC4409292; doi:10.1371/journal.pcbi.1004236)
Supplement: S5 Text — (PDF) [file pcbi.1004236.s017.pdf]

S5 Text: Evidence for chromatin modification-driven promoter cycling mechanism

Here we present the experimental evidence for histone modification driven promoter cycle summarized in Fig. 3B in the main text. Many histone modifications have been shown to contribute to modulation of binding affinity of cofactors to regulatory sites on chromatin [6]. For instance, during promoter activation, H4R3 methylation, mediated by the methylase PRMT1, increases the affinity of histone acetyltransferase p300 for chromatin. P300 primarily modifies several lysine residues on the tail of histone H4 [18]. Similarly, acetylation of histone H3 lysines by CBP enhances binding and activity of CARM1 that catalyzes H3R17 methylation [19]. Specific acetylation marks, such as H3K14Ac and H4K16Ac, increase the binding of activating chromatin remodeling complexes in various experimental systems [46, s20], while others (H3K9Ac, H4K12Ac and H4K8Ac) attract basal TFs, such as Bdf1 (yeast), TAF3 or TAF250 (mammals) [46-48]. Thus the approximate order of the modification events leading to promoter activation would be: histone arginine methylation, lysine methylation and ATP-dependent remodeling.

Methylation marks (H3K4me3, H3K79me3, H4K20me), previously thought to act exclusively in a repressive fashion, were found to associate with the TSSs of actively transcribing genes [18, s21, s22]. At least two of these are deposited by RNA polymerase II associated complexes, i.e. H3K4me3 by MLL/COMPASS (mammalian/yeast) and H3K79me3 by DOLT1 (mammalian) [23, 24]. These marks attract the chromatin remodeling complex ISW1 [s25], and the repressing complex Set3, which exhibits histone H3 and H4 deacetylation activity in yeast [s26-28]. In higher eukaryotes, similar findings have been reported. For instance, in *Drosophila* the remodeling complex Nurf and in human CHD1 have affinity for H3K4me3 [s29, s30] and for HDAC8, of which the enzymatic activity is enhanced in the presence of H4K20me1 [s31]. Therefore, at least a single act of transcription initiation would be required to add a methylation mark that can further lead to promoter de-activation by re-establishing repression of TSS by nucleosomes. DNA methylation may also play a role in promoter repression [23]. Transient DNA methylation occurs after RNA polymerase II binding, and one of the best-described repressive mammalian complexes, Mi-2/NurD, which contains chromatin remodeling and HDAC subunits, has domains with affinity for methylated CpG. This suggests that DNA methylation is coupled to the promoter de-activation process.

In order to re-start the transcription cycle the remaining modifications have to be removed. This requires an enzymatic activity that is distinct from that of the activity that put the modifications in place, removing lysine and arginine histone methylations. While the identity of most of these enzymes has been established [s32], less is known about their affinity for chromatin and the dependence of this affinity on histone modifications. For the H3K4 demethylase LSD1 it is suggested that deacetylation

of histones has to occur before it can bind successfully and another H3K4 demethylase, Lid, was shown to inhibit HDAC-dependent histone deacetylation [s33, s34]. The enzyme that removes arginine methylation has been found in higher eukaryotes, but its binding preference is unknown. If the acetylation indeed occurs before demethylation then three following orders of promoter reversion are possible: (i) deamination, deacetylation and demethylation, (ii) deacetylation, deamination and demethylation, (iii) deacetylation, demethylation and deamination. If we assume a strict sequential mechanism, we can exclude the 3rd one, as this would produce a singly arginine-methylated state equivalent to the first step of promoter activation (Fig. 3, main text). The other two orders are equally plausible based on current knowledge.
