# Supplementary material for: Multiplex Eukaryotic Transcription (In)activation: Timing, Bursting and Cycling of a Ratchet Clock Mechanism
Source: PLoS Comput Biol. 2015 Apr 24;11(4):e1004236. doi: 10.1371/journal.pcbi.1004236 (PMC4409292; doi:10.1371/journal.pcbi.1004236)
Supplement: S6 Text — (PDF) [file pcbi.1004236.s018.pdf]

S6 Text: Supporting Information References

s1. Hammar P, Wallden M, Fange D, Persson F, Baltekin O, Ullman G, et al. Direct measurement of transcription factor dissociation excludes a simple operator occupancy model for gene regulation. Nat Genet. 2014;46(4):405-8. doi: 10.1038/ng.2905 pmid: 24562187

s2. Peeters E, van Oeffelen L, Nadal M, Forterre P, Charlier D. A thermodynamic model of the cooperative interaction between the archaeal transcription factor Ss-LrpB and its tripartite operator DNA. Gene. 2013;524(2):330-40. doi: 10.1016/j.gene.2013.03.118 pmid: 23603352

s3. Taylor IC, Workman JL, Schuetz TJ, Kingston RE. Facilitated binding of GAL4 and heat shock factor to nucleosomal templates: differential function of DNA-binding domains. Genes & Development. 1991;5(7):1285-98. doi: 10.1101/gad.5.7.1285 pmid: 2065977

s4. Dobrzyński M, Bruggeman FJ. Elongation dynamics shape bursty transcription and translation. Proceedings of the National Academy of Sciences. 2009;106(8):2583-8. doi: 10.1073/pnas.0803507106 pmid: 19196995

s5. Berg OG, von Hippel PH. Diffusion-controlled macromolecular interactions. Annu Rev Biophys Biophys Chem. 1985;14:131-60. pmid: 3890878

s6. Ribbeck K, Gorlich D. Kinetic analysis of translocation through nuclear pore complexes. Embo J. 2001;20(6):1320-30. doi: 10.1093/emboj/20.6.1320 pmid: 11250898

s7. Braga J, Desterro JMP, Carmo-Fonseca M. Intracellular Macromolecular Mobility Measured by Fluorescence Recovery after Photobleaching with Confocal Laser Scanning Microscopes. Mol Biol Cell. 2004;15(10):4749-60. doi: 10.1091/mbc.E04-06-0496 pmid: 15292455

s8. Hinow P, Rogers CE, Barbieri CE, Pietenpol JA, Kenworthy AK, DiBenedetto E. The DNA Binding Activity of p53 Displays Reaction-Diffusion Kinetics. Biophys J. 2006;91(1):330-42. doi: 10.1529/biophysj.105.078303 pmid: 16603489

s9. Phair RD, Scaffidi P, Elbi C, Vecerova J, Dey A, Ozato K, et al. Global nature of dynamic protein-chromatin interactions in vivo: three-dimensional genome scanning and dynamic interaction networks of chromatin proteins. Mol Cell Biol. 2004;24(14):6393-402. doi: 10.1128/MCB.24.14.6393-6402.2004 pmid: 15226439

s10. Zhang X, Houk KN. Why enzymes are proficient catalysts: beyond the Pauling paradigm. Acc Chem Res. 2005;38(5):379-85. doi: 10.1021/ar040257s pmid: 15895975

s11. Carlsson B, Häggblad J. Quantitative Determination of DNA-Binding Parameters for the Human Estrogen Receptor in a Solid-Phase, Nonseparation Assay. Analytical Biochemistry. 1995;232(2):172-9. doi: 10.1006/abio.1995.0004 pmid: 8747472

s12. Luijsterburg MS, von Bornstaedt G, Gourdin AM, Politi AZ, Mone MJ, Warmerdam DO, et al. Stochastic and reversible assembly of a multiprotein DNA repair complex ensures accurate target site recognition and efficient repair. J Cell Biol. 2010;189(3):445-63. pmid: 20439997

s13. Mone MJ, Bernas T, Dinant C, Goedvree FA, Manders EM, Volker M, et al. In vivo dynamics of chromatin-associated complex formation in mammalian nucleotide excision repair. Proc Natl Acad Sci U S A. 2004;101(45):15933-7. doi: 10.1073/pnas.0403664101 pmid: 15520397

s14. Politi A, Mone MJ, Houtsmuller AB, Hoogstraten D, Vermeulen W, Heinrich R, et al. Mathematical modeling of nucleotide excision repair reveals efficiency of sequential assembly strategies. Mol Cell. 2005;19(5):679-90. doi: 10.1016/j.molcel.2005.06.036 pmid: 16137623

s15. Poux AN, Cebrat M, Kim CM, Cole PA, Marmorstein R. Structure of the GCN5 histone acetyltransferase bound to a bisubstrate inhibitor. Proc Natl Acad Sci U S A. 2002;99(22):14065-70. doi: 10.1073/pnas.222373899 pmid: 12391296

s16. Spencer TE, Jenster G, Burcin MM, Allis CD, Zhou J, Mizzen CA, et al. Steroid receptor coactivator-1 is a histone acetyltransferase. Nature. 1997;389(6647):194-8. doi: 10.1038/38304 pmid: 9296499

s17. Metivier R, Reid G, Gannon F. Transcription in four dimensions: nuclear receptor-directed initiation of gene expression. EMBO Rep. 2006;7(2):161-7. doi: 10.1038/sj.embor.7400626 pmid: 16452926

s18. Li X, Hu X, Patel B, Zhou Z, Liang S, Ybarra R, et al. H4R3 methylation facilitates {beta}-globin transcription by regulating histone acetyltransferase binding and H3 acetylation. Blood. 2010;115(10):2028-37. doi: 10.1182/blood-2009-07-236059 pmid: 20068219

s19. Daujat S, Bauer U-M, Shah V, Turner B, Berger S, Kouzarides T. Crosstalk between CARM1 Methylation and CBP Acetylation on Histone H3. 2002;12(24):2090-7. doi: 10.1016/S0960-9822(02)01387-8 pmid: 12498683

s20. Chandy M, Gutierrez JL, Prochasson P, Workman JL. SWI/SNF Displaces SAGA-Acetylated Nucleosomes. Eukaryotic Cell. 2006;5(10):1738-47. doi: 10.1128/ec.00165-06 pmid: 17030999

s21. Gerber M, Shilatifard A. Transcriptional Elongation by RNA Polymerase II and Histone Methylation. Journal of Biological Chemistry. 2003;278(29):26303-6. doi: 10.1074/jbc.R300014200 pmid: 12764140

s22. Guenther MG, Levine SS, Boyer LA, Jaenisch R, Young RA. A Chromatin Landmark and Transcription Initiation at Most Promoters in Human Cells. 2007;130(1):77-88. doi: 10.1016/j.cell.2007.05.042 pmid: 17632057

s23. Dreijerink KMA, Mulder KW, Winkler GS, Hoppener JWM, Lips CJM, Timmers HTM. Menin Links Estrogen Receptor Activation to Histone H3K4 Trimethylation. Cancer Res. 2006;66(9):4929-35. doi: 10.1158/0008-5472.can-05-4461 pmid: 16651450

s24. Steger DJ, Lefterova MI, Ying L, Stonestrom AJ, Schupp M, Zhuo D, et al. DOT1L/KMT4 Recruitment and H3K79 Methylation Are Ubiquitously Coupled with Gene Transcription in Mammalian Cells. Mol Cell Biol. 2008;28(8):2825-39. doi: 10.1128/mcb.02076-07 pmid: 18285465

s25. Santos-Rosa H, Schneider R, Bernstein BE, Karabetsou N, Morillon A, Weise C, et al. Methylation of Histone H3 K4 Mediates Association of the Isw1p ATPase with Chromatin. 2003;12(5):1325-32. doi: 10.1016/S1097-2765(03)00438-6 pmid: 14636589

s26. Couture J-Fo, Dirk LMA, Brunzelle JS, Houtz RL, Trievel RC. Structural origins for the product specificity of SET domain protein methyltransferases. Proceedings of the National Academy of Sciences. 2008;105(52):20659-64. doi: 10.1073/pnas.0806712105 pmid: 19088188

s27. Kim T, Buratowski S. Dimethylation of H3K4 by Set1 Recruits the Set3 Histone Deacetylase Complex to 5' Transcribed Regions. Cell. 2009;137(2):259-72. doi: 10.1016/j.cell.2009.02.045 pmid: 19379692

s28. Wang A, Kurdistani SK, Grunstein M. Requirement of Hos2 Histone Deacetylase for Gene Activity in Yeast. Science. 2002;298(5597):1412-4. doi: 10.1126/science.1077790 pmid: 12434058

s29. Clapier CR, Cairns BR. The Biology of Chromatin Remodeling Complexes. Annual Review of Biochemistry. 2009;78(1):273-304. doi: 10.1146/annurev.biochem.77.062706.153223 pmid: 19355820

s30. Flanagan JF, Mi L-Z, Chruszcz M, Cymborowski M, Clines KL, Kim Y, et al. Double chromodomains cooperate to recognize the methylated histone H3 tail. Nature. 2005;438(7071):1181-5. doi: 10.1038/nature04290 pmid: 16372014

s31. Gurard-Levin ZA, Mrksich M. The Activity of HDAC8 Depends on Local and Distal Sequences of Its Peptide Substrates. Biochemistry. 2008;47(23):6242-50. doi: 10.1021/bi800053v pmid: 18470998

s32. Wang Y, Wysocka J, Sayegh J, Lee Y-H, Perlin JR, Leonelli L, et al. Human PAD4 Regulates Histone Arginine Methylation Levels via Demethylimination. Science. 2004;306(5694):279-83. doi: 10.1126/science.1101400 pmid: 15345777

s33. Forneris F, Binda C, Vanoni MA, Battaglioli E, Mattevi A. Human Histone Demethylase LSD1 Reads the Histone Code. Journal of Biological Chemistry. 2005;280(50):41360-5. doi: 10.1074/jbc.M509549200 pmid: 16223729

s34. Lee N, Erdjument-Bromage H, Tempst P, Jones RS, Zhang Y. The H3K4 Demethylase Lid Associates with and Inhibits Histone Deacetylase Rpd3. Mol Cell Biol. 2009;29(6):1401-10. doi: 10.1128/mcb.01643-08 pmid: 1618865

s35. Schultz BE, Misialek S, Wu J, Tang J, Conn MT, Tahilramani R, et al. Kinetics and Comparative Reactivity of Human Class I and Class IIb Histone Deacetylases. Biochemistry. 2004;43(34):11083-91. doi: 10.1021/bi0494471 pmid: 15323567

s36. Thompson PR, Kurooka H, Nakatani Y, Cole PA. Transcriptional Coactivator Protein p300. Kinetic characterization of its histone acetyltransferase activity. J Biol Chem. 2001;276(36):33721-9. doi: 10.1074/jbc.M104736200 pmid: 11445580

s37. Yue WW, Hassler M, Roe SM, Thompson-Vale V, Pearl LH. Insights into histone code syntax from structural and biochemical studies of CARM1 methyltransferase. Embo J. 2007;26(20):4402-12. doi: 10.1038/sj.emboj.7601856 pmid: 17882261

s38. Darzacq X, Singer RH, Shav-Tal Y. Dynamics of transcription and mRNA export. Current Opinion in Cell Biology. 2005;17(3):332-9. doi: 10.1016/j.ceb.2005.04.004 pmid: 15901505

s39. Izban MG, Luse DS. Factor-stimulated RNA polymerase II transcribes at physiological elongation rates on naked DNA but very poorly on chromatin templates. J Biol Chem. 1992;267(19):13647-55. pmid: 1618865

s40. Mason PB, Struhl K. Distinction and Relationship between Elongation Rate and Processivity of RNA Polymerase II In Vivo. Molecular Cell. 2005;17(6):831-40. doi: 10.1016/j.molcel.2005.02.017 pmid: 15780939

s41. Uptain SM, Kane CM, Chamberlin MJ. Basic Mechanisms Of Transcript Elongation And Its Regulation. Annual Review of Biochemistry. 1997;66(1):117-72. doi: 10.1146/annurev.biochem.66.1.117 pmid: 9242904

s42. Polikanov YS, Bondarenko VA, Tchernaenko V, Jiang YI, Lutter LC, Vologodskii A, et al. Probability of the site juxtaposition determines the rate of protein-mediated DNA looping. Biophys J. 2007;93(8):2726-31. doi: 10.1529/biophysj.107.111245 pmid: 17573434
